# Calcium Metal Mechanochemically Defluorinates PFAS to Environmentally Benign CaF_2_

**DOI:** 10.1021/jacs.6c11635

**Published:** 2026-09-29

**Authors:** Luis Simbari, Moosa Wasim, Anže Zupanc, Shrestha Banerjee, Benjamin M. Gallant, Joshua Deakin, Francisco Alvarado Cesar, Roly J. Armstrong, Tomislav Friščić, Erli Lu, Dominik J. Kubicki

**Affiliations:** † School of Chemistry, 1724 University of Birmingham, Birmingham B15 2TT, U.K.; ‡ Birmingham Centre for Mechanochemistry and Mechanical Processing, University of Birmingham, Birmingham B15 2TT, U.K.; § Facility for Electron Microscopy, University of Birmingham, Birmingham B15 2TT, U.K.; ∥ School of Natural and Environmental Sciences, 5994 Newcastle University, Newcastle Upon Tyne NE1 7RU, U.K.

## Abstract

Fluoropolymers and other per- and polyfluoroalkyl substances (PFAS) are among the most persistent anthropogenic materials. Although bulk fluoropolymers such as polytetrafluoroethylene (PTFE) are often regarded as chemically inert, weathering and abrasion generate long-lived fluorinated microplastics, while molecular PFAS accumulate in the environment as mobile contaminants. Existing destruction strategies can cleave C–F bonds, but typically require incineration or aggressive reagents and frequently generate water-soluble fluoride products that remain environmentally problematic. Here we show that calcium metal enables solvent-free mechanochemical defluorination of PTFE and perfluorooctanoic acid (PFOA) at ambient starting temperature without external heating, while irreversibly sequestering fluorine as calcium fluoride, a nontoxic, insoluble, and environmentally benign solid. Quantitative solid-state ^19^F NMR, with detection limits explicitly assessed, shows no resolvable PTFE after 30 min of milling with Ca and Mg (<1.0% residual PTFE); Sr gives near-complete conversion (<10% residual PTFE), whereas Ba gives only partial (∼64% residual PTFE) conversion after 30 min of milling. TEM and SEM-EDX reveal CaF_2_-rich nanocrystalline domains intimately mixed with an oxygen-containing disordered carbonaceous material. By coupling efficient defluorination with fluoride sequestration, this work establishes a mechanochemical strategy for mineralization of persistent fluorinated materials without generating soluble alkali-metal fluoride waste as a secondary environmental hazard, while defining the analytical and process-safety constraints relevant to further development.

## Graphical abstract

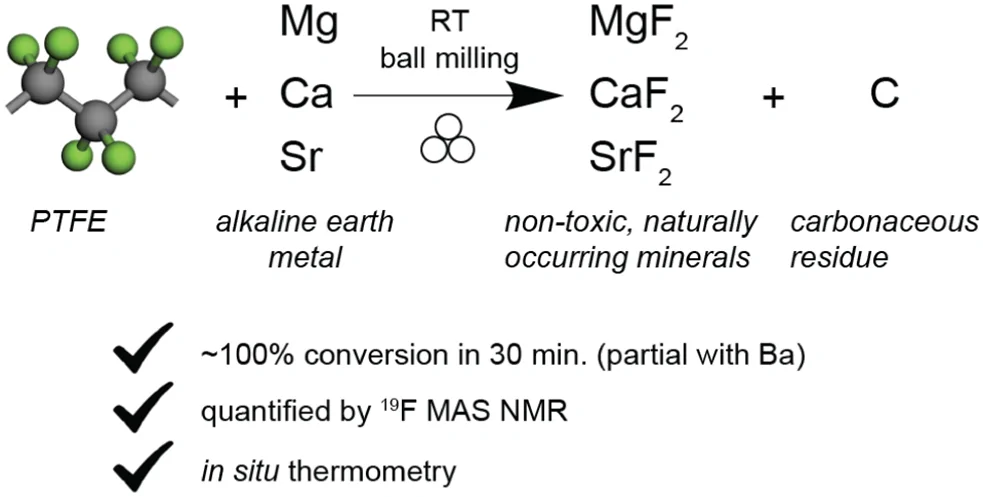

## Introduction

Fluoropolymers and other per- and polyfluoroalkyl substances (PFAS) owe their technological value and environmental persistence to strong C–F bonds, which make materials such as polytetrafluoroethylene (PTFE) chemically, thermally and biologically resistant, but also lead to long-lived fluorinated microplastics and mobile environmental contaminants.^1^ Existing destruction strategies can cleave C–F bonds but often require harsh conditions,^2–7^ aggressive reagents, solvents, long reaction times,^8,9^ or generate soluble fluoride products^10–14^ whose environmental fate remains problematic. Here we show that calcium metal enables rapid, solvent-free mechanochemical defluorination of PTFE and perfluorooctanoic acid at ambient starting temperature without external heating, while irreversibly sequestering fluorine as insoluble, nontoxic calcium fluoride. Quantitative solid-state ^19^F magic-angle spinning NMR shows complete conversion of PTFE fluorine, within the stated upper bound, into CaF_2_ within 30 min, with no soluble fluoride detected in the water extract. This work establishes calcium-mediated mechanochemistry as a strategy for PFAS destruction designed around both chemical efficiency and environmentally benign fluorine immobilization.

Fluorinated materials play a central role in modern technology owing to the exceptional strength of the carbon–fluorine bond. PTFE, one of the earliest, most widely used fluoropolymers, is emblematic of this stability and has been extensively characterized as an “extreme polymer” resistant to chemical, thermal, and biological degradation.^1^ While bulk PTFE is often considered environmentally inert, mechanical abrasion, aging, and weathering lead to fragmentation into fluorinated microplastics that persist indefinitely in nature.^15,16^ In parallel, per- and polyfluoroalkyl substances (PFAS) accumulate as mobile and bioactive contaminants in water and soil, creating a global environmental challenge.^17–19^

Numerous strategies have been explored to destroy or recycle fluorinated materials. Thermal pyrolysis and plasma-based approaches can induce C–F bond cleavage but require extreme temperatures and substantial energy input.^4–7^ Chemical routes based on molten bases, supercritical media, or highly aggressive reagents similarly suffer from harsh operating conditions and limited scalability.^2,3^ More recent chemical defluorination strategies have achieved PTFE and PFAS defluorination under milder conditions, but remain dependent on solvent-based media and prolonged reaction times of more than 10 h.^8,9^ Surface-level chemical defluorination of PTFE using reductants, radicals, or alkali metal atoms has been known for decades,^20,21^ but these reactions typically remain confined to thin interfacial layers and do not address bulk fluoropolymer persistence.

More recently, reductive strategies based on alkali metals have shown that bulk C–F bond cleavage in PTFE is achievable under comparatively mild conditions.^22^ Lithium-, sodium- and potassium-based approaches, including mechanochemical activation, efficiently convert PTFE into alkali metal fluorides and carbonaceous residues at or near room temperature, and some enable fluoride recovery for subsequent chemical upcycling.^10–14^ Related mechanochemical approaches have also been extended to molecular PFAS using bases, oxides or inorganic salts as co-milling agents.^23–25^

Metal-fluoropolymer mixtures have long been studied as highly energetic pyrolants and reactive materials, with fluoropolymer-to-metal fluoride conversion providing the thermodynamic driving force. Under milling, such systems can enter the broader regime of mechanically induced self-sustaining reactions, in which progressive comminution and interfacial activation are followed by a rapidly propagating reaction front and ignition. Reductive fluoropolymer mechanochemistry must therefore be interpreted not only as C–F activation but also in the context of this established energetic materials literature.^26–31^

However, these approaches do not resolve a key problem: the environmental fate of fluorine after defluorination. An ideal destruction strategy should not only cleave C–F bonds efficiently, but also convert fluorine into an intrinsically nontoxic, environmentally inert, and operationally safe end product. Alkali-metal systems fall short on this point because they generate soluble fluoride salts and rely on highly reactive reagents that impose substantial handling constraints. We therefore sought a mechanochemical reductant that could combine effective defluorination with benign fluoride sequestration and safety.

## Experimental Section

### Materials

Virgin PTFE was purchased from RS Components as a rod (length 500 mm, diameter 50 mm) and machined on a Colchester Triumph VS2500 lathe to produce PTFE swarf (thickness 0.8–1.2 mm, width 2–2.5 mm). Perfluorooctanoic acid (PFOA) (Sigma-Aldrich, 95%), Mg (Sigma-Aldrich, chips, 6–35 mesh, 99.98% trace metals basis), Ca (SLS, granules, 10–20 mm particle size, >98.5% trace metal basis), Sr (Fisher Scientific, granules, <19 mm, 99% trace metal basis), Ba (Merck, beads, 0.5–2.0 mm particle size, 99% trace metals basis) were used as received. Sr and Ba were stored in a nitrogen glovebox. All other reagents were stored in air. Accordingly, the as-received Ca granules are expected to bear air-formed Ca–O, Ca–OH and carbonate surface species.^32^ The thickness and composition of this native layer were not measured.

### PTFE and PFOA Degradation Reactions

All starting reagents were weighed out into stainless-steel (316 grade) 10 mL jars and one stainless steel (316 grade) ball (⌀10 mm, weight: 4.17 g) was added. The reaction mixtures were ground in an electric ball mill (Retsch Mixer Mill MM 400) for different amounts of time at 30 Hz, unloaded in air, and characterized with powder XRD and solid-state NMR. Reaction stoichiometries were calculated using the −CF_2_− repeating unit (1 mmol −CF_2_− = 50 mg) and assuming the formation of the corresponding metal fluoride. Safety precautions are summarised in Box [Boxed-text box1-fo] and Box [Boxed-text box2-fo].

1 **Safety note:**

Metal-fluoropolymer mixtures are energetic compositions and may undergo mechanically induced self-sustaining reactions. All experiments were therefore restricted to the stated small scale and conducted in closed, appropriately rated jars with the mill shield lowered. Remote operation and shielding are recommended during development. The absence of visible ignition for a given Ca or Sr run at this scale does not establish safety on scale-up. Any increase in scale requires reaction calorimetry, pressure/heat-release assessment and engineered control of heat and energy input.

2 **Safety note:**

Hydrogen is generated during mechanochemical reactions of acidic PFAS, such as PFOA, with reactive metals, and hydrogen-air mixtures are explosive. Strict exclusion of oxygen is therefore essential for safe operation.

The following amounts of reagents were used:

PTFE + Mg: 4 mmol (200 mg) PTFE + 4 mmol (97 mg) Mg (reagents:ball ratio = 1:14)

PTFE + Ca: 4 mmol (200 mg) PTFE + 4 mmol (160 mg) Ca (reagents:ball ratio = 1:12)

PTFE + Sr: 2 mmol (100 mg) PTFE + 2 mmol (175 mg) Sr (reagents:ball ratio = 1:15)

PTFE + Ba: 2 mmol (100 mg) PTFE + 2 mmol (275 mg) Ba (reagents:ball ratio = 1:11)

For the PTFE + Sr and PTFE + Ba reactions, 2 mmol was used to keep the reagents:ball ratio comparable to that in the other two reactions. For the liquid-assisted-grinding (LAG) test, Ca and PTFE (360 mg total solid stoichiometric mass) were milled with heptane (36 μL; η = 0.10 μL/mg; η is an empirical parameter defined as the volume of liquid additive (in μL) divided by the total mass of solid reactants (in mg) used during LAG).

When the PTFE + Mg reaction was conducted in a hermetically sealed screw-cap jar fitted with an air-tight gasket, it proceeded without visible incident. In contrast, when carried out in snap-on jars, the reaction produced an audible pop together with sparks and a flash of light within the mill enclosure, consistent with rapid ignition of the Mg/PTFE mixture (Box [Boxed-text box1-fo]). Accordingly, all PTFE + Mg experiments were restricted to small scale and must not be conducted in snap-on jars or scaled beyond the conditions described here. By contrast, the PTFE + Ca and PTFE + Sr reactions proceeded without visible incident in snap-on jars under the same milling conditions.

PFOA reactions were conducted in hermetically sealed 10 mL Retsch screw-cap jars fitted with a PTFE gasket and loaded in a nitrogen glovebox. After milling, internal overpressure consistent with hydrogen evolution was observed and released in a controlled manner. Accordingly, these reactions should not be performed in snap-on jars or under air, owing to the risk of hydrogen ignition or explosion (Box [Boxed-text box2-fo]).

The following amounts of reagents were used:

PFOA + Ca (1:7.5 molar ratio): 0.5 mmol (207 mg) PFOA + 3.75 mmol (150 mg) Ca (reagents:ball ratio = 1:12)

### Washing of the PTFE + Ca Reaction Product with D_2_O to Probe Water-Soluble Fluorine-Containing Species

The PTFE + Ca reaction product (∼150 mg) was recovered from the milling jar and washed with D_2_O (3 × 5 mL) in a 50 mL Falcon tube. After each washing step, the solid and liquid phases were separated by centrifugation at 10,000 rpm, and the supernatant was collected. The combined supernatants were concentrated on a rotary evaporator to a final volume of 0.5 mL and analyzed by ^19^F liquid-state NMR spectroscopy. The ^19^F NMR spectrum was recorded on a 9.4 T Bruker Avance III spectrometer using 16 scans and a recycle delay of 2 s.

### Powder X-Ray Diffraction (PXRD)

PXRD was carried out using Cu K_α_ X-rays on a Panalytical Empyrean diffractometer using a step size of 0.018° and a total acquisition time of 1 h per pattern.

### Scanning Electron Microscopy and Energy-Dispersive X-Ray Spectroscopy (SEM-EDX)

Ca/PTFE products collected after 5, 15, and 30 min were examined on a Thermo Fisher Scientific Apreo microscope. EDX maps were acquired at 10 kV with 768 × 512 pixels and total acquisition times of 1196–1213 s. Pixel-wise Ca/F and Ca/O colocalization was evaluated from the raw spectral maps as described in Supplementary Note 3 and Figure S13.

### Transmission Electron Microscopy (TEM)

TEM and HRTEM images were recorded on a JEOL JEM-2100 transmission electron microscope at an accelerating voltage of 200 kV. Pixel sizes were calibrated from the embedded scale bars. The calibration, window-selection and quality-control criteria are described in Supplementary Note 4.

### Solid-State NMR

Solid-state ^19^F (376.4 MHz) and ^13^C (100.6 MHz) Magic Angle Spinning (MAS) NMR spectra were recorded on a Bruker Avance Neo 9.4 T spectrometer equipped with a 3.2 mm MAS probe, except for the ^13^C spectrum of the PTFE + Sr reaction, which was carried out using a 4 mm MAS probe. The powder samples were packed into 3.2 mm zirconia rotors, which were typically spun at 15 or 20 kHz using dry nitrogen gas. ^13^C chemical shifts were referenced using the ^13^C −CH_2_– peak of solid adamantane as a secondary reference.^33^ ^19^F was referenced indirectly, based on the ^13^C reference, through the unified Ξ scale in accordance with the IUPAC recommendation.^34^ ^19^F *T*_1_ saturation-recovery measurements were used to determine the optimum recovery for quantitative spectra. In a few cases where recovery was incomplete, the intensities and the corresponding LOD and LOQ values were corrected before fluorine fractions were calculated (Supplementary Note 2, Figure S2, and Table S1). The complete isotropic and sideband manifolds were included in quantitative integrations. No ^1^H or ^19^F decoupling was used for the PTFE + Mg, PTFE + Ca, and PTFE + Sr reaction products, as it was found that it has no effect on the line widths. The ^13^C spectrum of neat PFOA was recorded using ^19^F–^13^C cross-polarization (CP), using the following RF strengths: 98 kHz (^19^F contact pulse), 70 kHz (^13^C contact pulse), and 85 kHz (^19^F decoupling). Further acquisition parameters are given in Table S2. In some spectra, we observed additional signal broadening, which we attribute to bulk magnetic susceptibility effects (Supplementary Note 1).^35^

### Quantification of ^19^F MAS NMR Spectra

Spectra were baseline-corrected and normalized to the total observed^19^F signal. Where signals were sufficiently resolved, the relative abundance of each fluorine-containing species was determined by integrating its isotropic resonance and associated spinning sidebands; overlapping contributions were quantified by spectral deconvolution. Residual PTFE at low abundance was evaluated using a spectrum-specific detection-limit analysis. Briefly, the metal fluoride signals and spinning sidebands near the expected PTFE isotropic resonance were fitted together with a possible PTFE contribution, and the fitted PTFE peak height was compared with the intensity fluctuations in signal-free regions of the spectrum. This noise threshold was converted into a PTFE fraction using the relationship between PTFE isotropic peak height and independently integrated total PTFE intensity in spectra acquired at the same MAS rate. Limits of detection (LOD) and quantification (LOQ) were defined as 3 and 10 times the estimated noise, respectively. Non-detections are therefore reported as upper bounds rather than as zero. Further details are provided in Supplementary Note 2 and Figures S16–S17.^36,37^

### In Situ Temperature Monitoring

Grinding jar temperature was monitored using the Elitech ICT-220 dual-thermocouple thermometer equipped with a K-type thermocouple. Each thermocouple was attached to the outer surface of the jar using electrical tape such that the probe remained in direct contact with the metal wall and in the same position for every experiment. The temperatures of the reaction jar and the empty reference jar were recorded simultaneously. The temperatures were recorded at 1 second intervals with a temperature resolution of 0.1 °C. The experimental setup is shown in Figure S14.

## Results and Discussion

### Mechanochemical Defluorination of PTFE

Inspired by recent sodium-based defluorination strategies,^10,11^ we hypothesized that a reaction with calcium could deliver a more favorable environmental outcome. Calcium provides a strong thermodynamic driving force for fluoride formation, but its fluoride product, CaF_2_, is sparingly soluble, nontoxic, and widely present in nature as the mineral fluorspar. Fluorspar is the main mineral feedstock for hydrofluoric acid production, which in turn is the primary feedstock for virtually all fluorine-bearing chemicals. We therefore reasoned that mechanochemical activation of calcium could combine efficient C–F bond cleavage and irreversible sequestration of fluorine in an environmentally inert mineral phase, in contrast to strategies that generate soluble fluoride salts. Such an approach could enable recovery of fluorine in a form compatible with the existing fluorochemical value chain, notably now that the US DOE identifies fluorspar as a critical mineral and projects rapidly increasing demand from battery technologies within the next decade.^38^

Ball milling PTFE with calcium metal leads to rapid chemical transformation at ambient starting temperature without external heating (Figure 1). Because calcium can be handled in air, the reaction proceeds without the requirement of working under inert gas conditions, as is the case for alkali metals. PTFE reflections were strongly attenuated and CaF_2_ reflections appeared after 15 min. Residual PTFE was not resolved by PXRD after 30 min (Figure S5). Quantitative solid-state ^19^F MAS NMR shows complete loss of CF_2_ resonances characteristic of PTFE, and the appearance of a single fluorine signal at −108 ppm corresponding to CaF_2_, after 30 min of milling (Figure 2A), representing an approximately 30–140-fold reduction in reaction time relative to recently reported solvent-based protocols.^8,9^ Rigorous quantification puts the 3σ upper bound of residual PTFE in two independent runs at <0.5% and <0.3% of observed fluorine. Qualitatively, no substantial amounts of soluble fluoride species are detected in the ^19^F liquid-state NMR of the water extract of the reaction products (Figure S7). The ^13^C MAS NMR spectra reveal extensive reorganization of the carbon framework, indicating that defluorination is accompanied by substantial carbon–carbon restructuring rather than superficial surface fluorine abstraction (Figure 2B). We attribute the broad ^13^C envelope centered near 110 ppm to an oxygen-containing, predominantly sp^2^-rich, disordered carbonaceous material. Its chemical shift is lower compared to many conventional carbon blacks and does not support a unique graphitic or molecular structure.^39,40^ The weak contribution at ca. 160 ppm may include oxygen-substituted aromatic and/or carbonyl/carboxyl environments. There is no resolved amorphous carbon halo in the XRD patterns of the materials, which are dominated by crystalline CaF_2_ (Figure S6).

**Figure 1:**
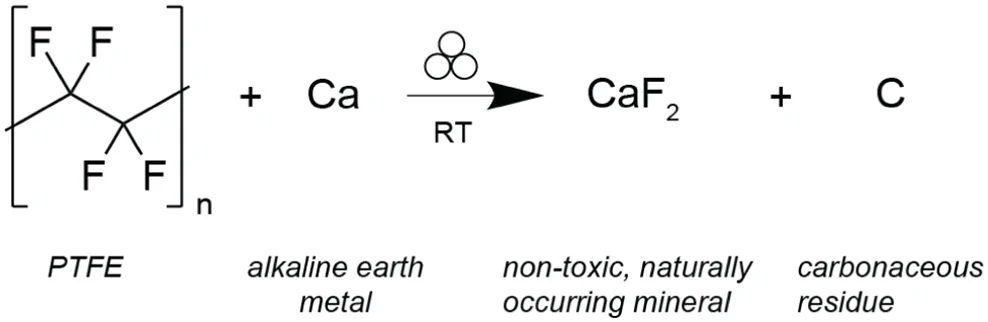
Schematic representation of PTFE defluorination by Ca metal. Other possible alkaline earth metals include Mg and Sr, which yield MgF_2_ and SrF_2_, respectively.

**Figure 2:**
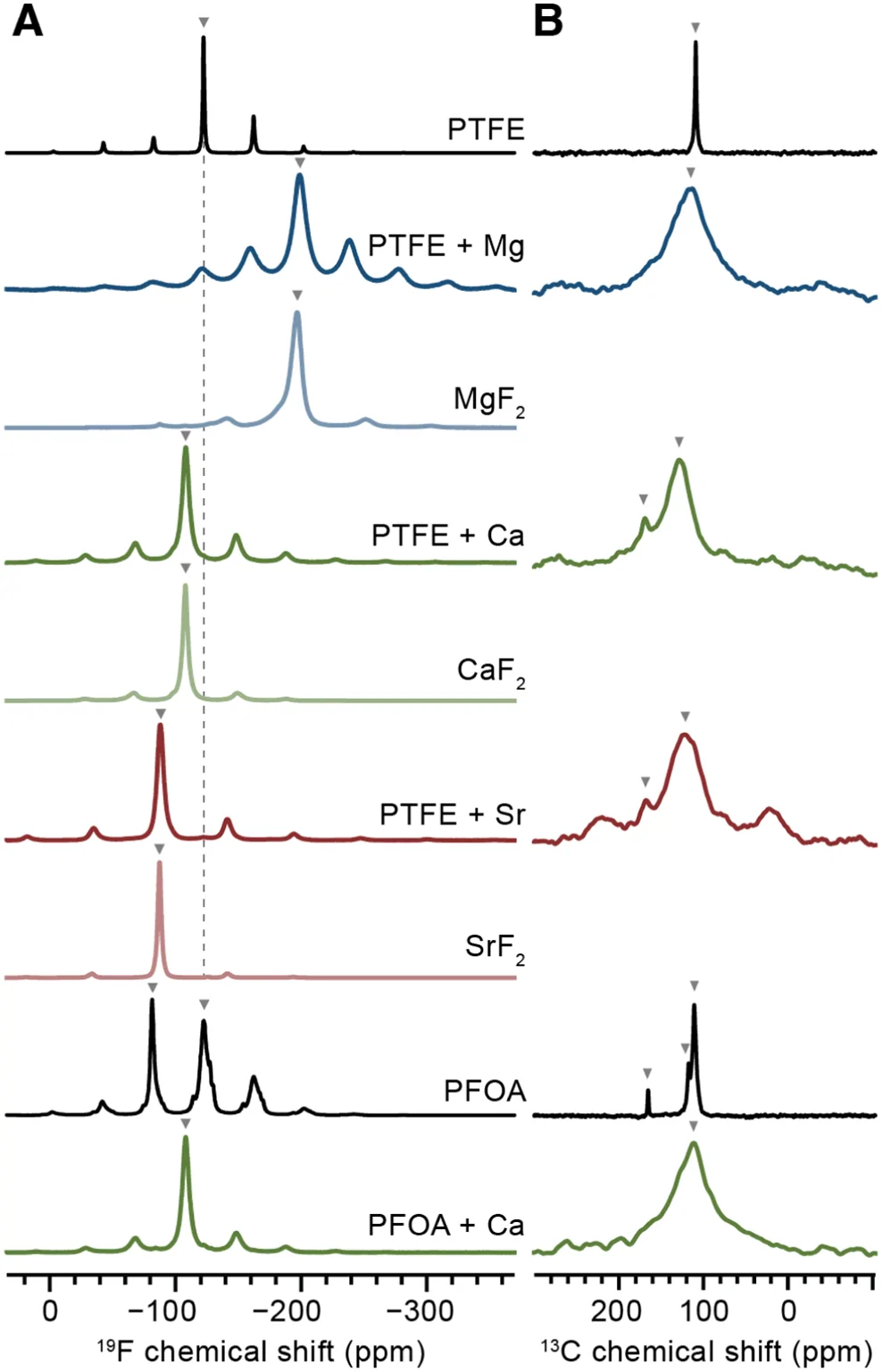
Mechanochemical defluorination of PFAS by alkaline-earth metals monitored by solid-state NMR: (A) ^19^F MAS NMR spectra of pristine PTFE, PTFE milled with Mg, Ca, or Sr, corresponding alkaline-earth metal fluoride references (MgF_2_, CaF_2_, SrF_2_), pristine perfluorooctanoic acid (PFOA), and PFOA milled with Ca. Gray triangle markers indicate isotropic peaks; all other peaks are spinning sidebands. (B) Corresponding ^13^C MAS NMR spectra highlighting changes in the polymer backbone upon mechanochemical reaction. Additional spectra and acquisition details are provided in Figures S1–S2 and Tables S1–S3.

The TEM images of products after 5, 15, and 30 min of milling show dense nanocrystalline domains embedded in lower-contrast submicrometer aggregates, reminiscent of a CaF_2_-carbon nanocomposite (Figure S10). Lattice fringes of ca. 0.30–0.31 nm consistent with CaF_2_(111) are present at the 15 and 30 min mark. SEM-EDX shows essentially no Ca/F pixel correlation at 5 min (Pearson r = −0.05) but strong Ca/F correlation at 15 and 30 min (r = 0.95 and 0.93), together with Ca/O correlation throughout (r = 0.48, 0.89 and 0.89; Figures S11–S13). These data show that the CaF_2_ and carbon phases are intimately mixed and are best thought of as a nanocomposite. Indeed, in a recent work on the reactivity of Na metal with PTFE, we have found that efficient extraction of the water-soluble NaF required milling with water,^11^ presumably to break up the nanocomposite. We anticipate that similar considerations would apply for the downstream processing of our reaction products.

We have also carried out the Ca/PTFE reaction in the presence of liquid heptane (LAG with η = 0.10 μL/mg) and the ^19^F MAS spectrum of the product showed essentially full conversion to CaF_2_ (3σ upper bound of 0.54% of observed fluorine) (Figure S9). This result is interesting in that it shows that a small amount of hydrocarbon does not prevent the reaction from proceeding efficiently, which might have implications, for example, for processing PTFE waste contaminated with machine oil.

### Extension to Molecular PFAS

Mechanochemical degradation of molecular PFAS has attracted increasing attention, particularly using co-milling agents. These studies demonstrate that mechanochemistry can facilitate C–F bond cleavage in otherwise inert perfluoroalkyl chains, but often results in complex fluoride speciation or only partially defluorinated intermediates.^23–25^ To determine whether calcium metal mechanochemistry extends beyond polymeric systems, we examined perfluorooctanoic acid (PFOA), a widely studied and environmentally relevant PFAS. Milling PFOA with calcium metal results in near-complete disappearance of characteristic perfluoroalkyl signatures in ^19^F MAS NMR spectra (Figure 2A,B). After 30 min, conservative upper bounds obtained at the relatively isolated PFOA CF_3_ position are 4.3% and 6.7% of observed fluorine for two independent reactions, corresponding to disappearance of at least 95.7% and 93.3% of PFOA, respectively. As in the case of PTFE, the product spectra are dominated by CaF_2_, and the ^13^C spectrum shows the broad oxygen-containing, largely sp^2^ resonance of disordered carbon described above, indicative of extensive C–F bond scission. The ability of calcium metal to induce defluorination in both polymeric and molecular systems shows that calcium-mediated mechanochemistry extends beyond PTFE to a molecular PFAS.

### Comparative Reactivity of Alkaline-Earth Metals toward PTFE

Magnesium-PTFE mixtures are well known as energetic compositions and are widely used as pyrolants, where ignition can be triggered by heat, friction, or impact.^26,28^ Motivated by the observation that Ca-PTFE reactions proceed reproducibly under mechanochemical conditions without ignition or runaway behavior, we set out to compare the reactivity of magnesium, calcium, strontium and barium toward PTFE under identical milling conditions.

First, we quantified reaction progress ex situ using ^19^F MAS NMR spectroscopy as a function of milling time. Three of the metals show complete (Mg, Ca) or near-complete (Sr) conversion of PTFE within 30 min. More precisely, no PTFE resonance is resolved for Mg within a conservative 1.0% resolution-limited bound or for Ca within <0.5% and <0.3% 3σ bounds for two independent runs. Sr retained 3.2% and 7.4% PTFE after 30 min while barium proved substantially less reactive with only approximately 36% fluorine present as BaF_2_ and the remainder as unreacted PTFE (Figure S8).

Next, we used in situ temperature monitoring using a thermocouple attached to the grinding jars. The three reaction systems show substantial differences in thermal behavior under otherwise identical milling conditions (Figure 3). Magnesium generates the largest thermal response (average temperature increase, Δ*T*, of 19 °C; average time integrated temperature rise, ∫Δ*Tdt*, of 207 °C·min), followed by calcium (Δ*T* = 16 °C, ∫Δ*Tdt* = 177 °C·min), and strontium (Δ*T* = 10 °C, ∫Δ*Tdt* = 175 °C·min). These observations suggest that there is a hierarchy of reactivity within this alkaline-earth series, namely, Mg is more reactive than Ca, which is more reactive than Sr. This trend cannot be rationalized by thermodynamic considerations alone, as the standard enthalpies of formation of MgF_2_, CaF_2_, and SrF_2_ are comparable (−1124, −1226, and −1217 kJ/mol, respectively) and so are the corresponding entropic contributions.^41^ Instead, we attribute the differences to kinetic control under mechanochemical conditions likely arising from the differences in mechanical properties of the three metals. The onset of the exothermic event follows the opposite order to reactivity, with Mg exhibiting the longest time-to-peak (13.2 min on average), followed by Ca (6.6 min) and Sr (4.6 min). We note that this induction delay correlates with both Mohs hardness and Young’s modulus, with Mg being the hardest and stiffest of the three metals and Sr the softest and least stiff (Figure S15). We observe no correlation with Poisson’s ratio, a measure of the coupling between axial and lateral strain, or the Pilling-Bedworth ratio, a measure of the extent to which an oxide layer can passivate a metal surface.^42,43^

**Figure 3:**
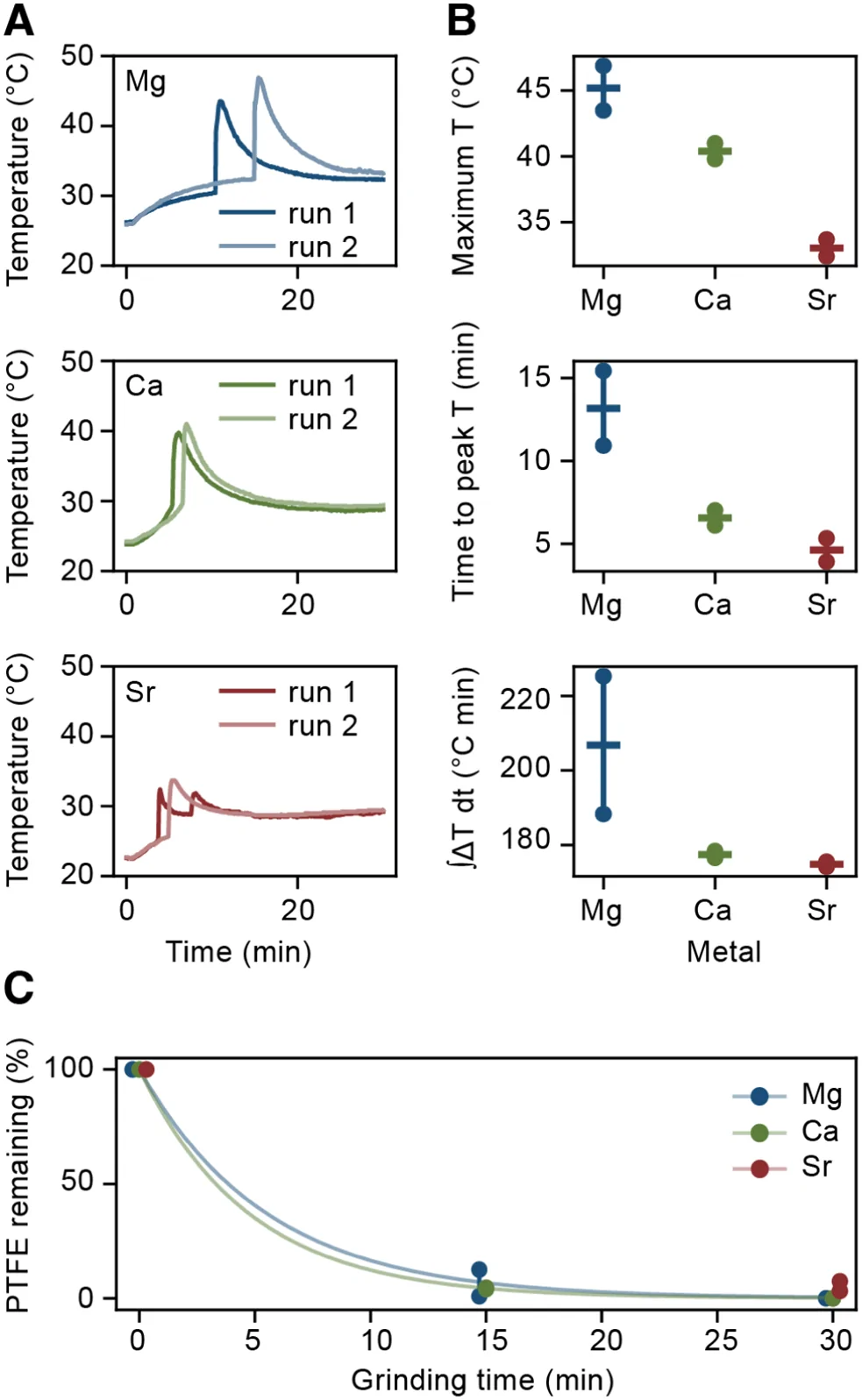
Mechanochemical defluorination of PFAS by alkaline-earth metals monitored by in situ thermometry and ex situ conversion monitoring as a function of grinding time: (A) In situ temperature profiles recorded during ball milling—metal-dependent thermal responses. (B) Summary of thermal metrics obtained from the temperature traces: peak temperature reached, time-to-peak temperature, and time-integrated temperature rise. (C) Residual PTFE content as a function of grinding time. Individual points are independently milled reactions. Lines are guides to the eye. Further spectra, numerical values underlying all panels, and NMR acquisition and quantification details are provided in Figures S1–S4, Tables S1–S5 and Supplementary Note 2.

The mechanochemical reaction products MgF_2_, CaF_2_, and SrF_2_ are all sparingly soluble in water (130, 16, and 1170 mg/L, respectively, at RT), which means that fluoride immobilization is favorable across this alkaline-earth metal series. The particularly low solubility of CaF_2_ means that calcium can be considered as the most effective alkaline-earth metal for fluoride immobilization. Under the conditions used here, Ca combines rapid defluorination with a smaller external jar-temperature rise than Mg, but the absence of visible ignition should not be taken as evidence of intrinsic process safety. Calcium therefore occupies an intermediate kinetic regime in which defluorination happens efficiently without the excessive heat generation observed for Mg. In analogy with mechanical alloying behavior, impact is expected to deform and fracture the metal, disrupt native surface films and create new reagent interfaces, which provide a plausible route for overcoming surface passivation.^44–46^ We propose that electron transfer from mechanically activated alkaline-earth metal surfaces into antibonding orbitals of C–F bonds promotes reductive cleavage, while formation of CaF_2_ sequesters fluorine and prevents recombination or formation of secondary fluorinated products.

The 10 mL jar experiments discussed here show chemical feasibility of this process at laboratory scale. Other mechanochemical reactions have been translated to continuous kilogram-per-hour extrusion, and PFAS degradation has been demonstrated in a 267 L industrial horizontal ball mill; commercial modular mechanochemical reactors are also being developed for waste treatment.^47–50^ These precedents show that mechanical processing can be scaled. However, because the present fluorochemical-metal mixtures can undergo mechanically induced self-sustaining reactions, we anticipate that scale-up will require finding new ways to tame the present chemistry.

In conclusion, our work establishes a solvent-free strategy for destroying persistent fluorinated materials while producing environmentally inert end products. We achieve this outcome by explicitly prioritizing non-toxicity and fluorine immobilization by judicious choice of the defluorination reagents: metallic Ca, Mg and Sr. Ca provides the distinctive advantage of combining rapid defluorination reactivity with controlled energy release and formation of CaF_2_, an insoluble, nontoxic, and environmentally benign fluorine sink, which is also the main industrial precursor to HF production. This makes calcium uniquely attractive for safer and operationally simple mechanochemical redox defluorination, while providing a blueprint for a circular fluorine economy. Although the systems studied here are PTFE and PFOA, the underlying chemistry targets the fluorinated carbon framework and we anticipate that it will be applicable to fluoropolymers and small-molecule PFAS more broadly. Our results show that mechanochemical redox chemistry can be designed around both performance and environmental compatibility.

## Supplementary Material



## Notes

Solid-state NMR acquisition details and relaxation data; additional NMR spectra and detection-limit analysis; PXRD patterns; in situ milling temperature data; SEM-EDX elemental maps and colocalization analysis; TEM images (PDF). Raw NMR, PXRD and microscopy data are available on Zenodo: https://zenodo.org/records/22027169 (DOI:10.5281/zenodo.22027169)
