# Supplementary material for: Calcium Metal Mechanochemically Defluorinates PFAS to Environmentally Benign CaF2
Source: J Am Chem Soc. 2026 Sep 29;148(40):43262–8. doi: 10.1021/jacs.6c11635 (PMC13674247; doi:10.1021/jacs.6c11635)
Supplement: Supplementary file 1 [file ja6c11635_si_001.pdf]

Supplementary Information

Calcium metal mechanochemically defluorinates PFAS to environmentally benign CaF_2

Luis Simbari,[1†] Moosa Wasim,[1†] Anže Zupanc,[1,2†] Shrestha Banerjee,[1] Benjamin M. Gallant,[1] Joshua Deakin,[1] Francisco Alvarado Cesar,[3] Roly J. Armstrong,[4] Tomislav Friščić,[1,2] Erli Lu,[1,2*] Dominik J. Kubicki[1*]

[1]*School of Chemistry, University of Birmingham; Birmingham, B15 2TT, UK*
[2]*Birmingham Centre for Mechanochemistry and Mechanical Processing, University of Birmingham, B15 2TT, UK*
[3]*Facility for Electron Microscopy, University of Birmingham; Birmingham, B15 2TT, UK*
[4]*School of Natural and Environmental Sciences, Newcastle University, Newcastle Upon Tyne NE1 7RU, UK*

*Corresponding author email: e.lu@bham.ac.uk, d.j.kubicki@bham.ac.uk

†These authors contributed equally

SAFETY NOTE: Mixtures of PTFE or PFOA with metals are energetic and must be handled with caution. Ball milling may initiate a mechanically induced self-sustaining reaction, creating a risk of rapid heat release and thermal runaway. PFOA mixtures with metals may additionally evolve flammable H_2 and generate pressure. All reactions reported here were conducted at a total reactant scale of <1 g using the precautions described in the main text. **These conditions should not be assumed to be transferable to larger scales.**

Supplementary Note 1. Bulk magnetic susceptibility effects in solid-state MAS NMR spectra

In some runs, for example PTFE + Mg, we observed pronounced spinning sidebands that were more intense than in the neat reference MgF_2. We attribute these to bulk magnetic susceptibility effects caused by jar abrasion leading to the presence of trace amounts of stainless steel particles and/or transition metal ions inside the sample that distort the homogeneity of the magnetic field, leading to an increase in anisotropy, as measured by sideband intensity. We found this effect to be small and not correlated to the specific reaction system or grinding time. We did not observe appreciable Paramagnetic Relaxation Enhancements (PREs), which would be expected if the paramagnetic species were incorporated into the structure of the inorganic fluorides.

Supplementary Note 2. Limits of detection (LOD) and limits of quantification (LOQ)

Each baseline-corrected spectrum was divided by its total ^{19}F integral between +180 and -480 ppm, so the resulting PTFE fractions describe the fraction of the observed fluorine signal. In a local window around the expected PTFE isotropic resonance, a quadratic baseline, pseudo-Voigt profiles for the nearest metal fluoride signals and SSBs, and a PTFE component centered at the expected position were fitted simultaneously. The fitted PTFE peak height therefore represents intensity remaining at the PTFE position after the overlapping metal fluoride contributions have been accounted for.

The noise level was estimated from nominally signal-free points between the sidebands, after excluding points within +/-8 ppm of every expected PTFE or metal fluoride signal. A robust estimate was used to prevent isolated spikes from dominating the calculation:

$$\sigma_{noise} = 1.4826\, median(|I_j - median(I)|).$$

Here, I_j is the normalized intensity of an individual signal-free data point. The factor 1.4826 puts the median absolute deviation on the same scale as a conventional standard deviation for normally distributed noise. Where too few signal-free points were available, the residuals between the fitted and experimental local spectra were used instead.

To convert this intensity noise into a PTFE fraction, an internal response factor was obtained at each MAS rate. For spectra in which PTFE was clearly detected, its isotropic peak height (h_i) was divided by the PTFE fraction (f_i) determined independently by integrating the complete PTFE isotropic + SSB pattern. The median of these ratios defined the response factor k_r for MAS rate r:

$$k_r = median_i(\frac{h_i}{f_i}).$$

$$f_{LOD,j} = \frac{3\sigma_j}{k_r}, \quad f_{LOQ,j} = \frac{10\sigma_j}{k_r}.$$

The 3σ and 10σ values define the limits of detection (LOD) and quantification (LOQ), respectively. Values below the LOD were reported as upper bounds rather than zero.

For the PFOA/Ca experiment (run 2), the conservatively integrated CF_3 region accounted for 1.33% of the total observed ^{19}F signal. Because the CF_3 group contains three of the 15 fluorine atoms in PFOA, this value was multiplied by 15/3=5, giving a relaxation-corrected upper bound of 6.7%. This corresponds to disappearance of at least 93.3% of the initial PFOA signature. Run 1 was quantified accordingly and corresponds to the disappearance of at least 95.7% of the initial PFOA.

Where the recycle delay used to acquire a spectrum for quantitative analysis did not provide complete longitudinal recovery for every species, the integrated intensities were corrected before the species fractions were calculated. For each species, the fully recovered intensity is obtained by dividing the observed intensity by its fractional recovery at the acquisition recycle delay. The recovery-corrected PTFE fraction was therefore calculated as

$$f_{PTFE,corr} = \frac{\frac{f_{PTFE,obs}}{R_{PTFE}(d_1)}}{\frac{f_{PTFE,obs}}{R_{PTFE}(d_1)} + \frac{1 - f_{PTFE,obs}}{R_{MF2}(d_1)}}.$$

The same transformation was applied to the spectrum-specific LOD and LOQ values by replacing $f_{PTFE,obs}$ with the corresponding uncorrected LOD or LOQ. Here, $R_s(d_1)$ is the fractional recovery of species s at the recycle delay d_1 used for acquisition. It was obtained from the fitted stretched-exponential saturation-recovery curve:

$$R_s(d_1) = 1 - exp[-(\frac{d_1}{T_{1,s}})^{\beta_s}].$$

Here, $T_{1,s}$ and β_s are the fitted characteristic recovery time and stretching exponent for species s, respectively; $\beta = 1$ corresponds to monoexponential recovery. The metal fluoride was generally the more slowly recovering component, such that its fractional recovery was lower than that of PTFE at the acquisition recycle delay. Incomplete recovery therefore suppresses the observed intensity of the metal fluoride product and biases the uncorrected PTFE fraction upward. Accordingly, the correction decreases the calculated residual PTFE fraction and the associated LOD and LOQ values; where the two recoveries were comparable, its effect is correspondingly negligible.

Supplementary Note 3. SEM–EDX map processing and colocalization analysis

Raw pixel-resolved EDX spectra were integrated after local background subtraction. Peak/background windows were O Kα 475–575 eV (background 415–465 and 585–635 eV), F Kα 630–725 eV (575–620 and 735–780 eV), and Ca Kα 3610–3780 eV (3440–3560 and 3830–3950 eV). Each elemental intensity was divided by total X-ray yield, smoothed with a 0.14 µm Gaussian kernel and square-root transformed for display. Pearson coefficients were calculated on the normalized map pixels to assess spatial coincidence. In the additive Ca/F overlays, magenta Ca plus green F becomes pale/white where both normalized channels are intense.

Supplementary Note 4. TEM morphology and HRTEM lattice fringe analysis

Pixel sizes were calculated from the embedded acquisition scale bars. Lattice analysis used fixed, non-overlapping 512×512 pixel windows in the 400–600 kx images. After Gaussian high-pass filtering ($\sigma = 20$ pixels), a two-dimensional Hann window was applied and Fourier amplitudes were searched between 2.55 and 5.65 nm^{-1}. Maxima were refined by a weighted centroid and checked independently by directional autocorrelation. A window was accepted when the normalized peak score was ≥1.55, the FFT- and autocorrelation-derived spacings differed by no more than the larger of 0.025 nm and 9% of the FFT-derived spacing, and the FFT contained a discrete centrosymmetric pair of maxima.

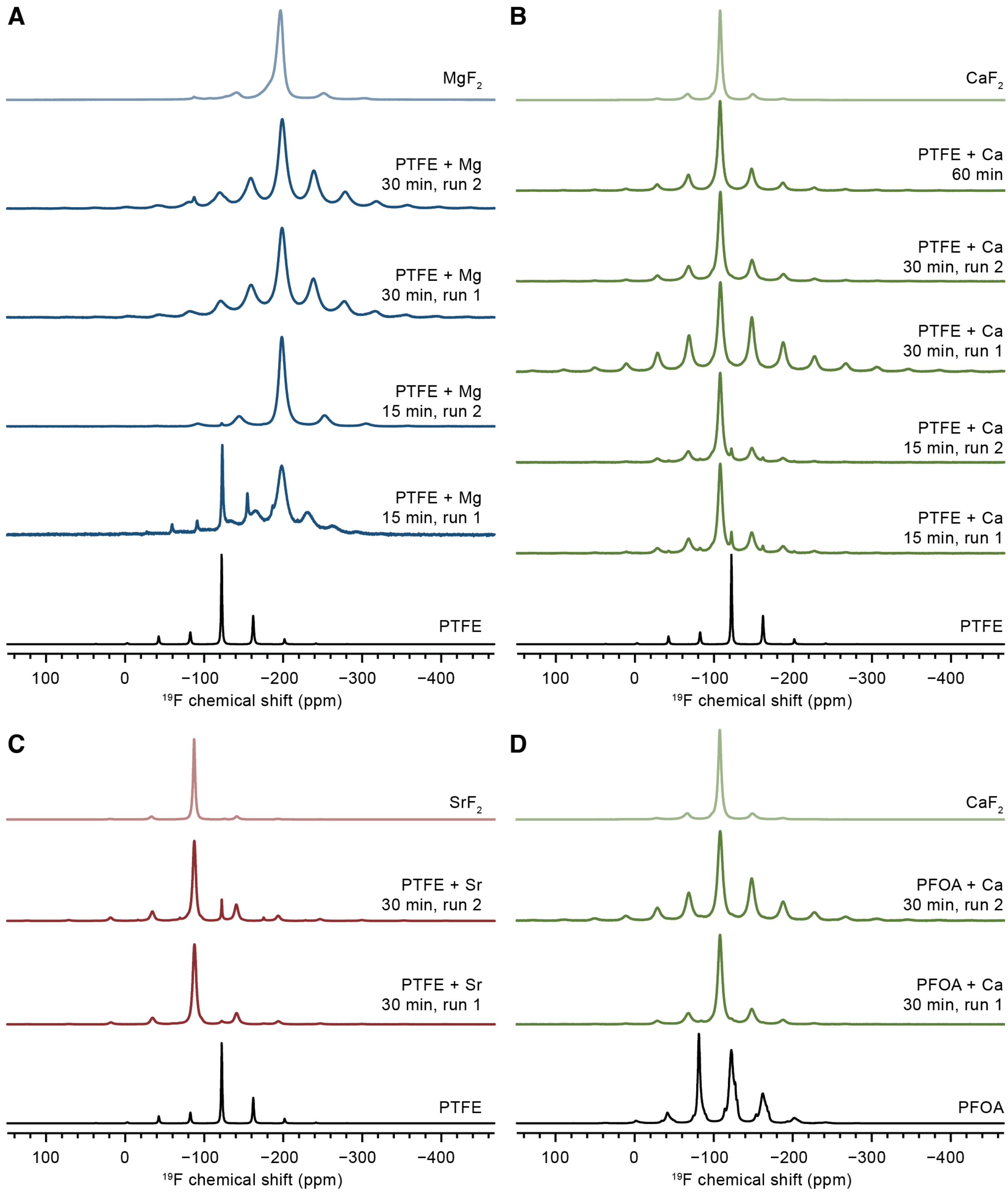

Figure S1. Quantitative ^{19}F MAS NMR datasets for different grinding times and replicates. (A) PTFE + Mg, (B) PTFE + Ca, (C) PTFE + Sr, (D) PFOA + Ca.

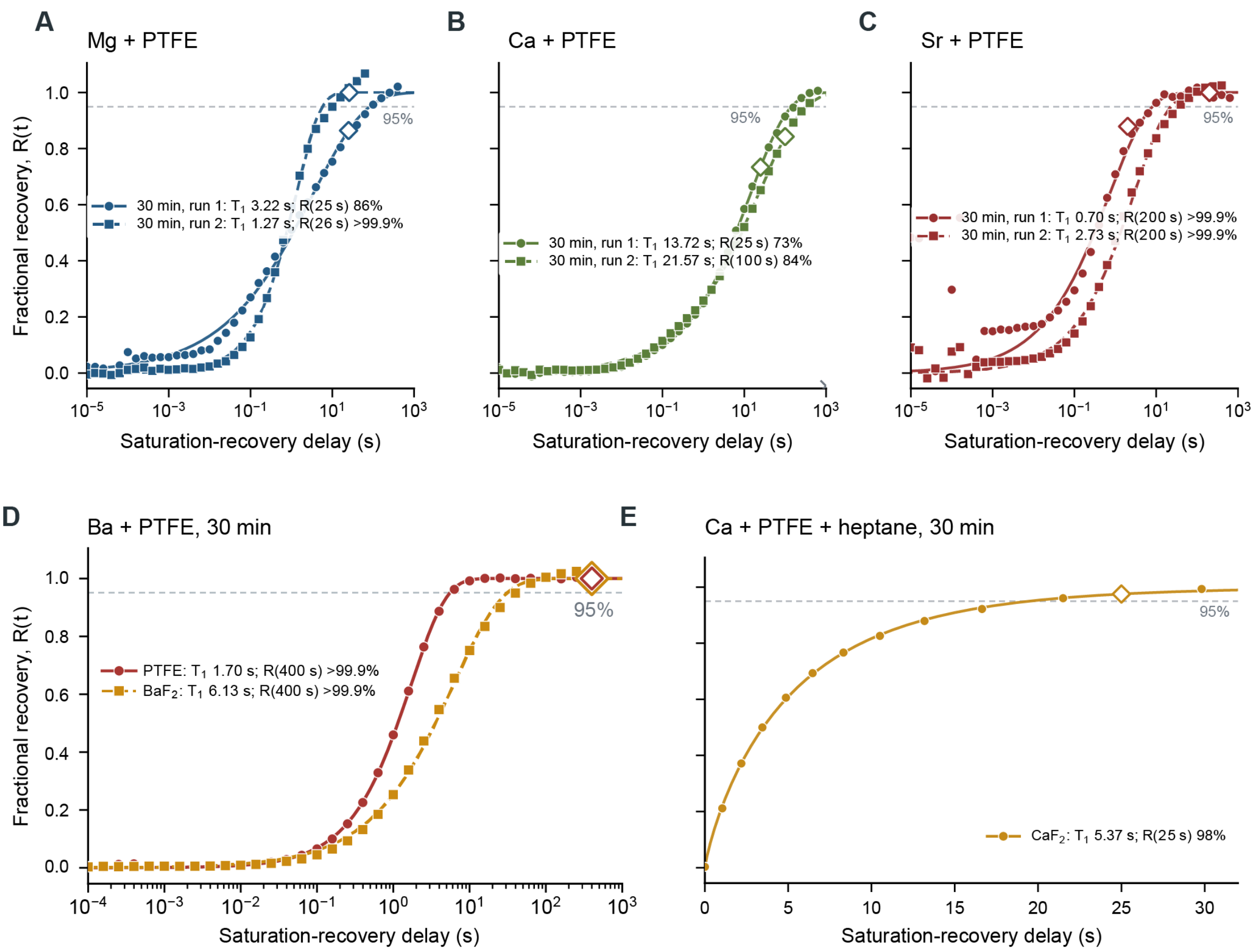

Figure S2. ^{19}F MAS NMR saturation-recovery curves measured on the products of the 30 min reaction runs (A) Mg + PTFE (for MgF_2), (B) Ca + PTFE (for CaF_2), (C) Sr + PTFE (for SrF_2), (D) Ba + PTFE (for BaF_2 and PTFE), (E) Ca + PTFE (for CaF_2) in the heptane-assisted Ca/PTFE reaction. Symbols are measured integrated intensities and curves are stretched-exponential fits with the fitted T_1 indicated in the legend. Open diamonds indicate the recycle delay used for the corresponding one-dimensional spectrum used for quantification. Horizontal dashed lines indicate 95% recovery.

Table S1. Summary of the fitted values obtained from the ^{19}F MAS NMR saturation-recovery curves in Figure S2.

Panel	Sample	Species	Fitted T_1 (s)	Fitted β	Recycle delay used (s)	Recovery at delay used (%)
A	Mg + PTFE, 30 min, run 1	MgF_2	3.2	0.34	25	86
A	Mg + PTFE, 30 min, run 2	MgF_2	1.3	0.72	26	100
B	Ca + PTFE, 30 min, run 1	CaF_2	13.7	0.47	25	73
B	Ca + PTFE, 30 min, run 2	CaF_2	21.6	0.40	100	84
C	Sr + PTFE, 30 min, run 1	SrF_2	0.7	0.45	200	100
C	Sr + PTFE, 30 min, run 2	SrF_2	2.7	0.52	200	100
D	Ba + PTFE, 30 min	PTFE	1.7	0.93	400	100
D	Ba + PTFE, 30 min	BaF_2	6.1	0.68	400	>99.9
E	Ca + PTFE + heptane, 30 min	CaF_2	5.4	0.85	25	98

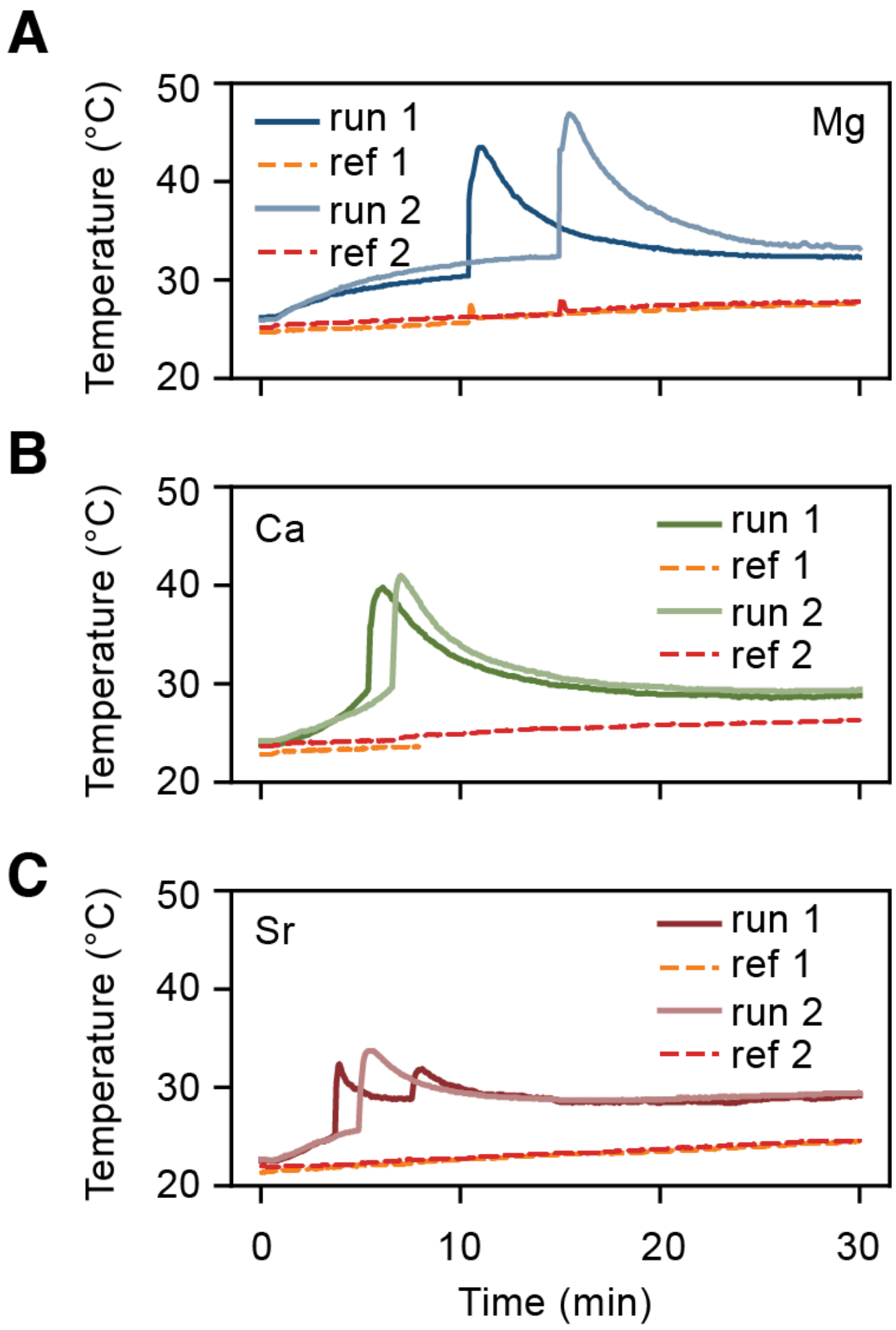

Figure S3. In situ temperature monitoring during mechanochemical reactions including empty reference jar measurements. In situ temperature profiles recorded during milling of PTFE with (A) Mg, (B) Ca, and (C) Sr. For each system, two independent batches are shown (run 1 and run 2), together with corresponding reference measurements (ref 1 and ref 2) obtained simultaneously from an empty milling jar mounted on the second arm of the mixer mill as a measure of the background temperature within the mixer mill enclosure. The reference trace for ref 1 in the PTFE + Ca experiment is truncated because of thermocouple failure during the measurement. We observed frequent thermocouple failure under the milling conditions used because of mechanical fatigue of the thermocouple wires. The data shown represent the most complete internally consistent dataset.

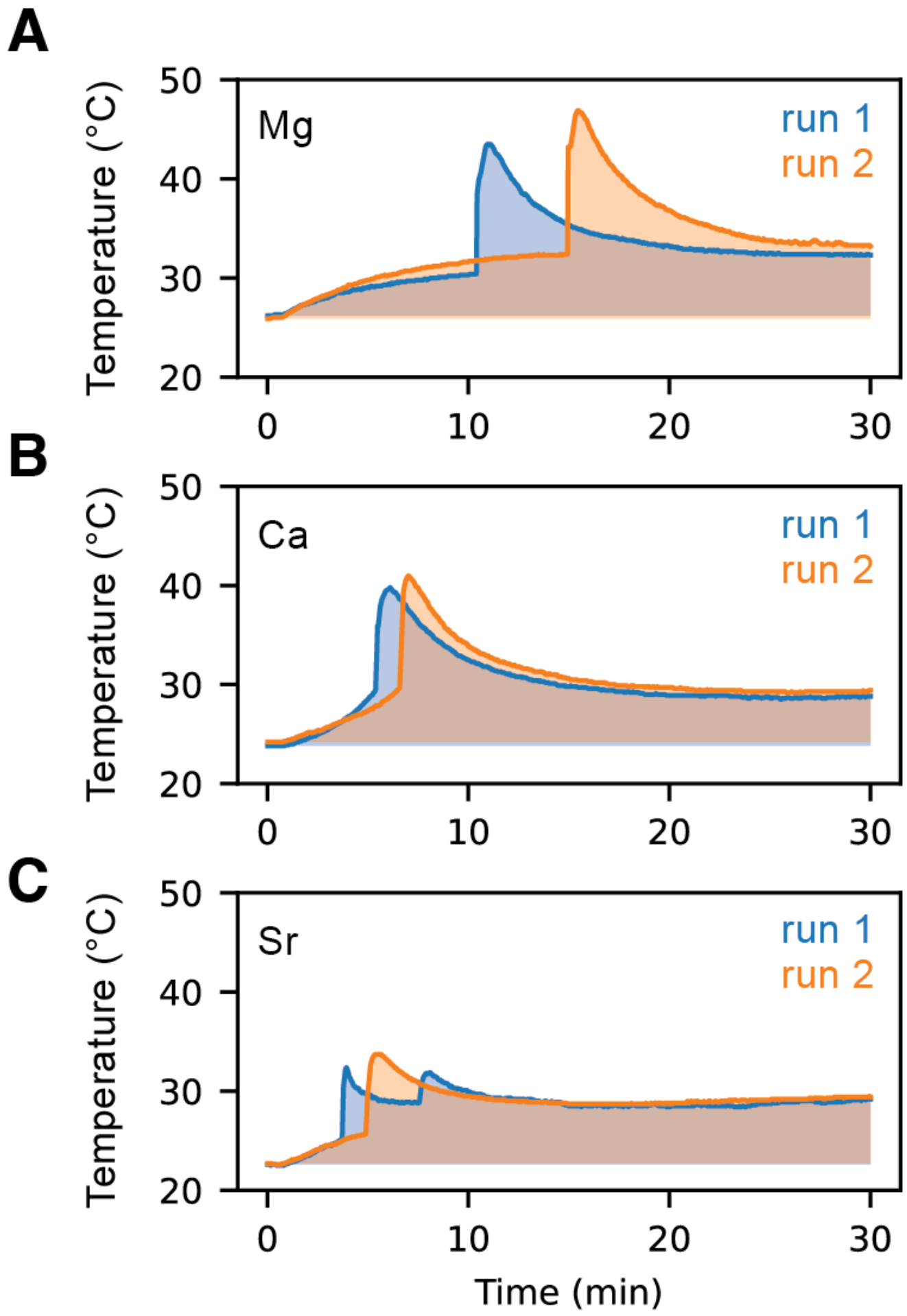

Figure S4. Integrated areas under the temperature curves (∫ΔT dt) for the in situ temperature profiles recorded during milling of PTFE with (A) Mg, (B) Ca, and (C) Sr. Baseline temperature is taken as the median of the first 60 seconds of each run, before milling was started. This initial 60 second period was used to establish a stable baseline for subsequent integration. The values are reported in Table S5.

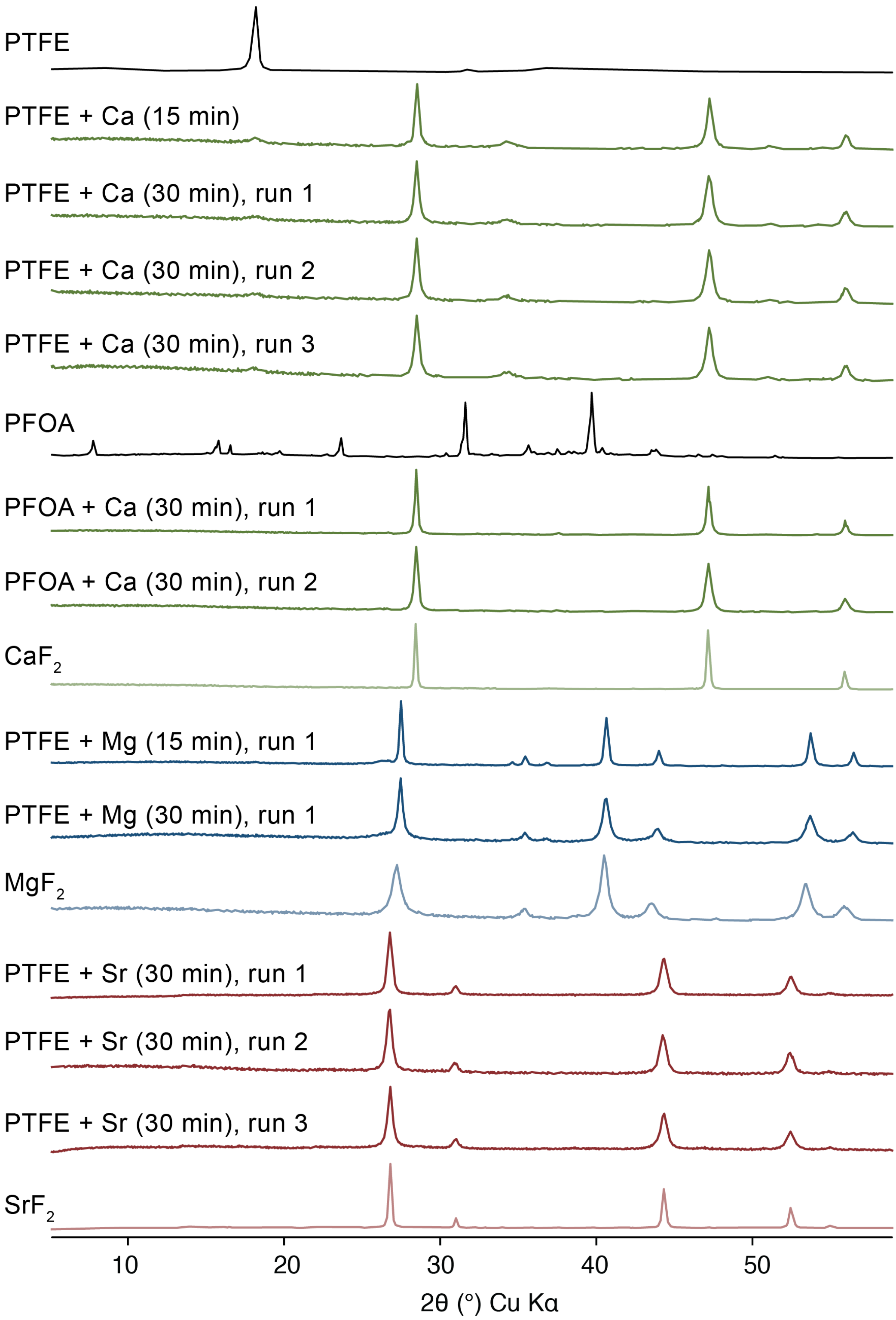

Figure S5. Powder XRD patterns of the reaction mixtures and reference metal fluorides, PTFE and PFOA. No baseline correction was applied.

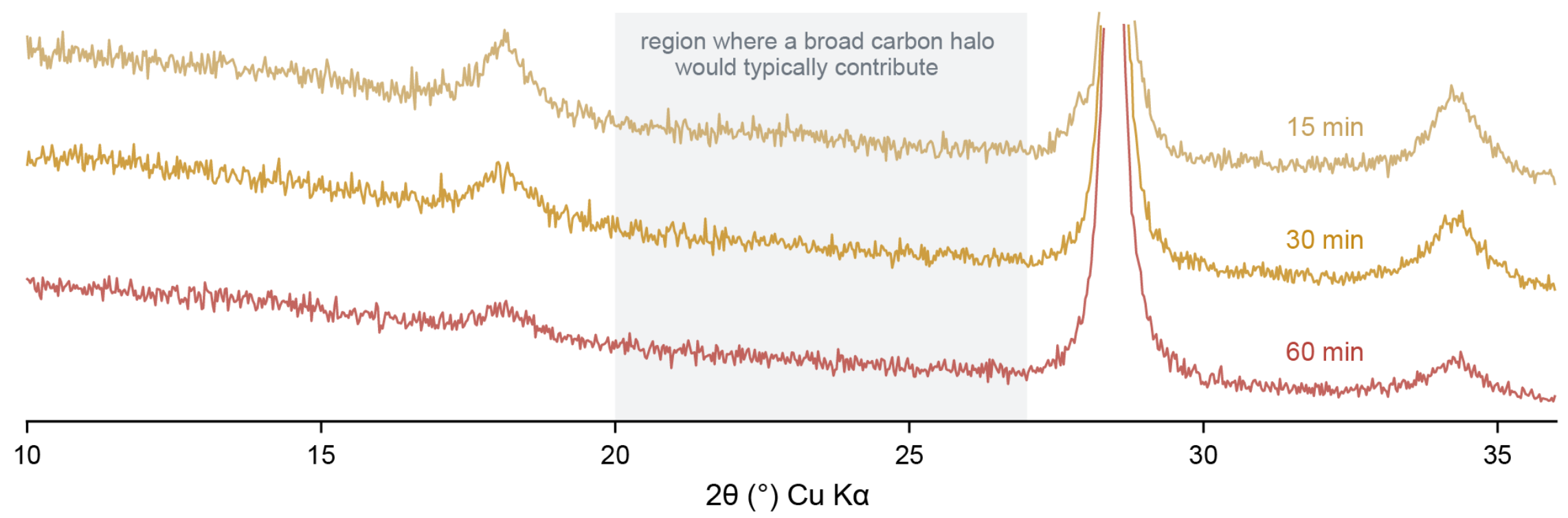

Figure S6. Low-angle range of the Ca/PTFE reaction PXRD patterns. No baseline correction was applied. The shaded region shows where amorphous carbon signal would typically appear.

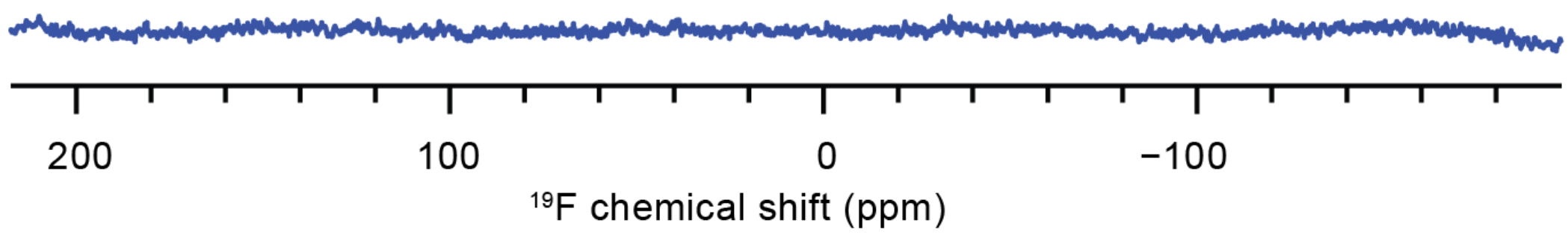

Figure S7. ^{19}F liquid-state NMR spectrum of the D_2O extract of the 30 min PTFE + Ca product (16 scans; 2 s recycle delay). No ^{19}F signal was observed under these conditions, which we take as a qualitative indication that there are no substantial amounts of dissolved fluorine-containing species. We note that the most appropriate technique for quantifying trace (ppb/ppt) quantities of PFAS is validated isotope-dilution LC-MS/MS.

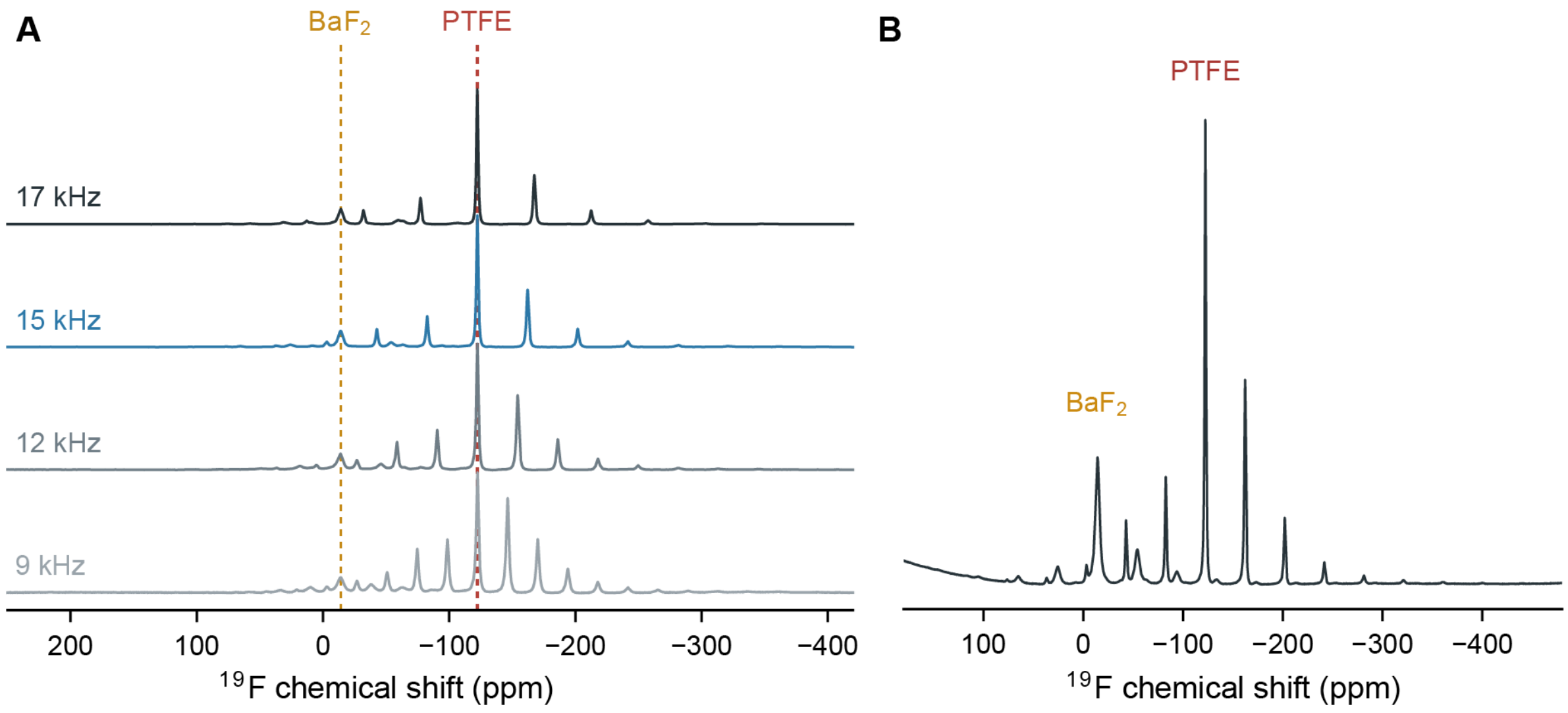

Figure S8. ^{19}F MAS NMR characterization of the Ba + PTFE reaction after 30 min of milling. (A) Variable MAS on the product of a preliminary substoichiometric reaction run (2 mmol -CF_2- + 1 mmol Ba) to identify the isotropic peak of BaF_2, and (B) a quantitative spectrum on the product of a stoichiometric reaction run (2 mmol -CF_2- + 2 mmol Ba). Recycle delay of 400 s. Quantification of the stoichiometric reaction in panel B gives approximately 36% of observed fluorine as BaF_2 and approximately 64% as residual PTFE.

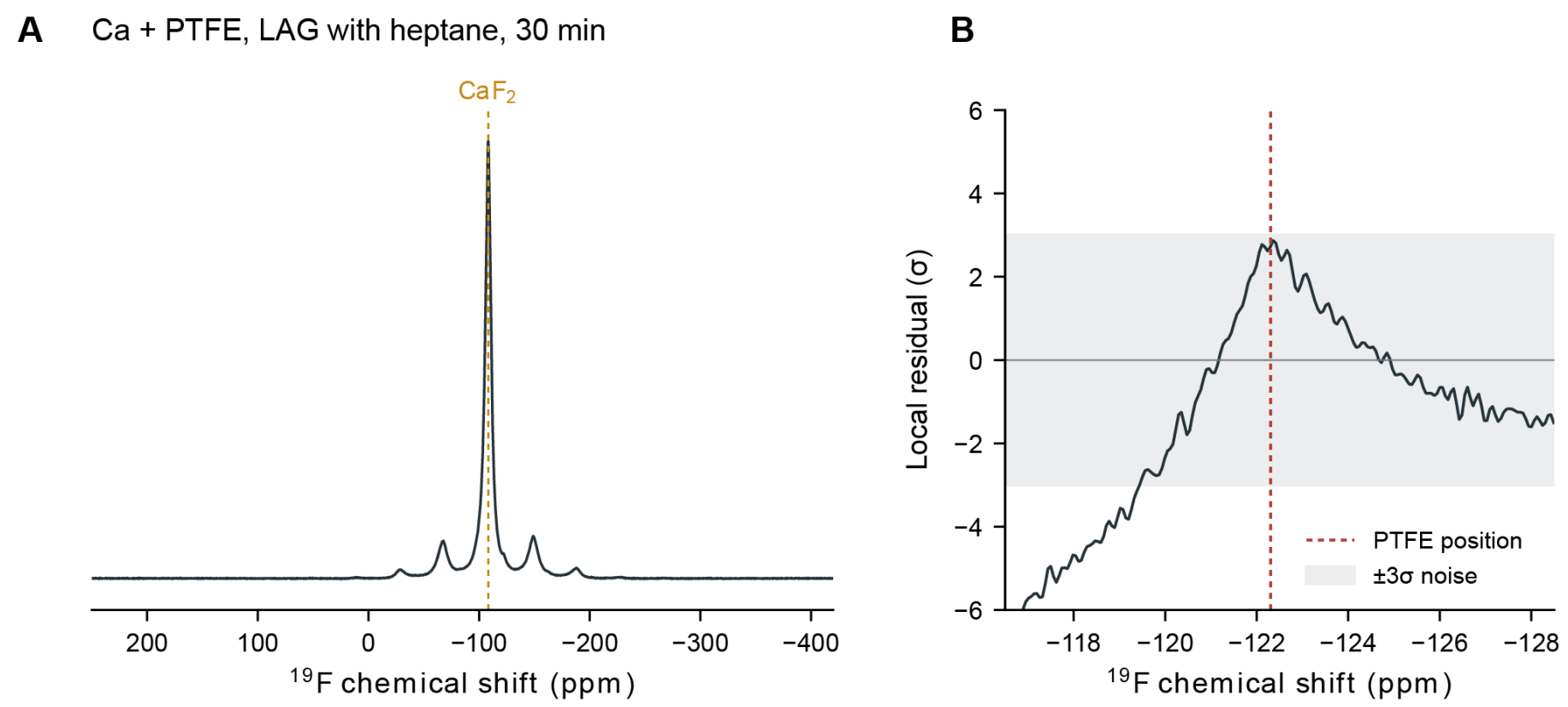

Figure S9. (A) Quantitative ^{19}F MAS NMR of product obtained in the Ca/PTFE reaction with liquid-assisted grinding with heptane ($\eta = 0.10$ µL/mg). (B) The residual at the PTFE position is 2.7 times the local residual noise standard deviation, below the 3σ detection criterion; LOD and LOQ are 0.54% and 1.80% of observed fluorine.

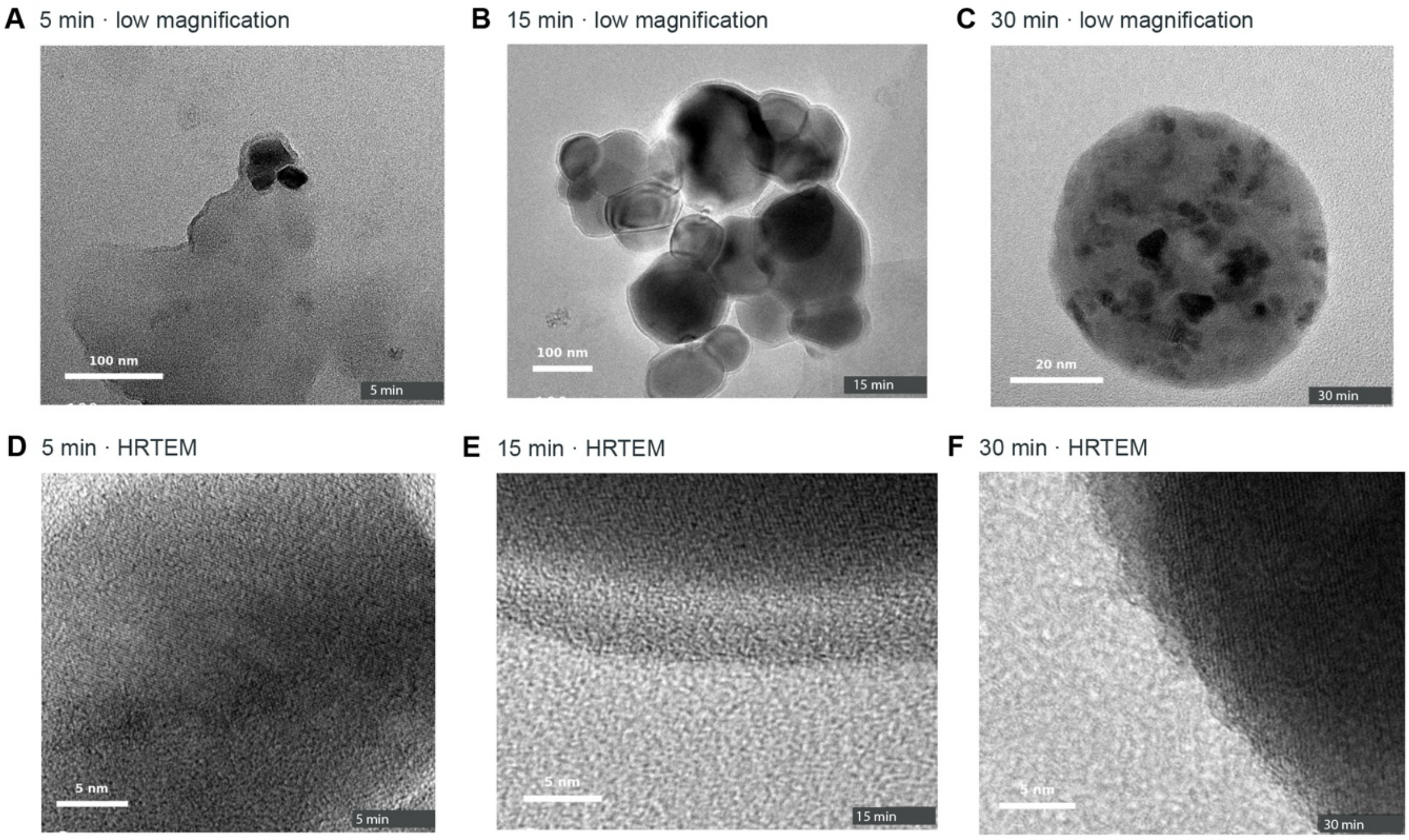

Figure S10. TEM/HRTEM micrographs of the Ca/PTFE reactions after 5, 15 and 30 min of milling. Dense crystalline domains are apparent, which are embedded within a lower-contrast matrix.

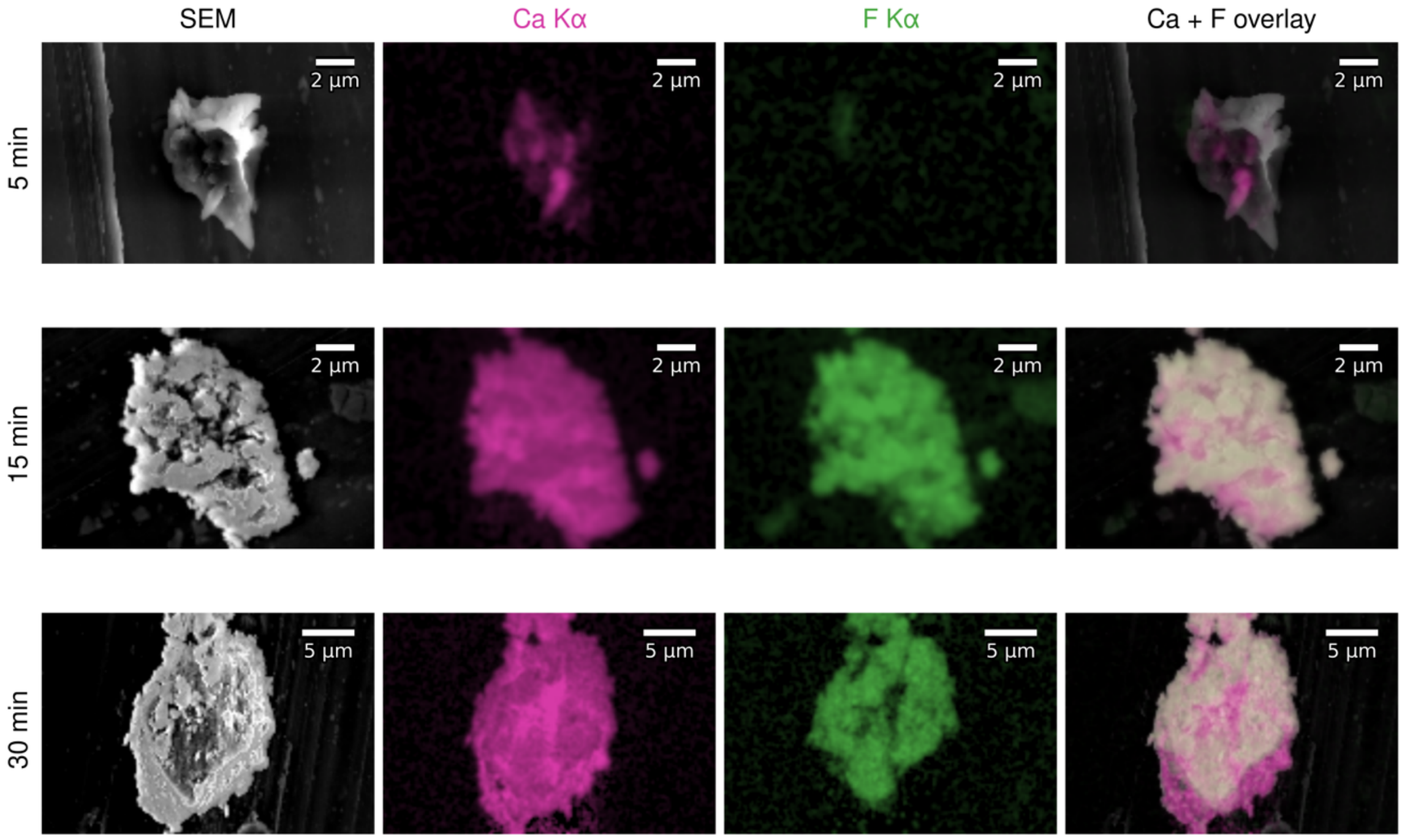

Figure S11. SEM-EDX maps of the Ca/PTFE reactions after 5, 15 and 30 min of milling. Ca and F channels and additive overlays are shown, which evidence a strong coincidence of Ca and F at 15 and 30 min. Pale/white overlay pixels indicate simultaneous high normalized intensities of Ca and F.

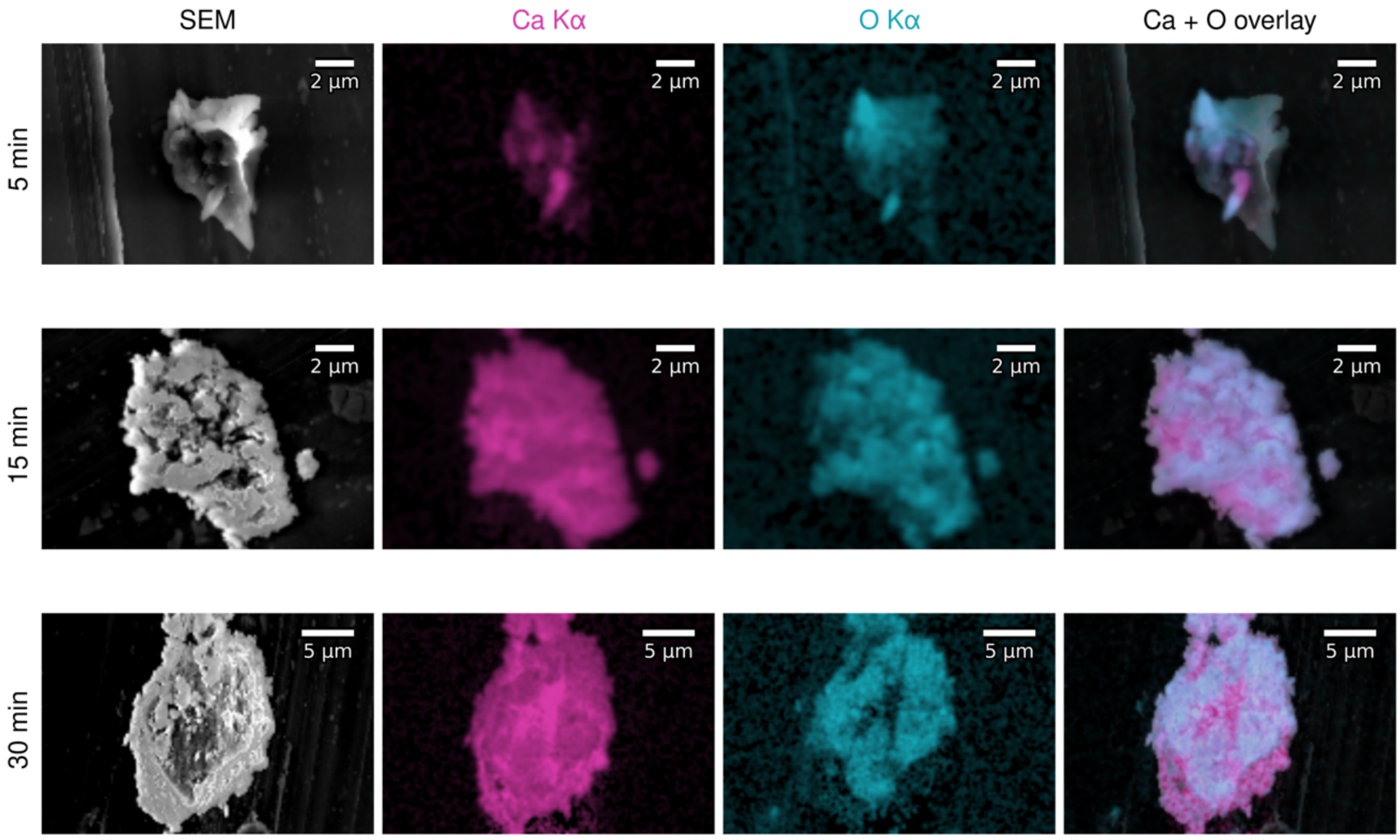

Figure S12. SEM-EDX maps of the Ca/PTFE reactions after 5, 15 and 30 min of milling. Ca and O channels and additive overlays are shown. Ca/O colocalization is moderate at 5 min and strong at 15 and 30 min (Figure S13).

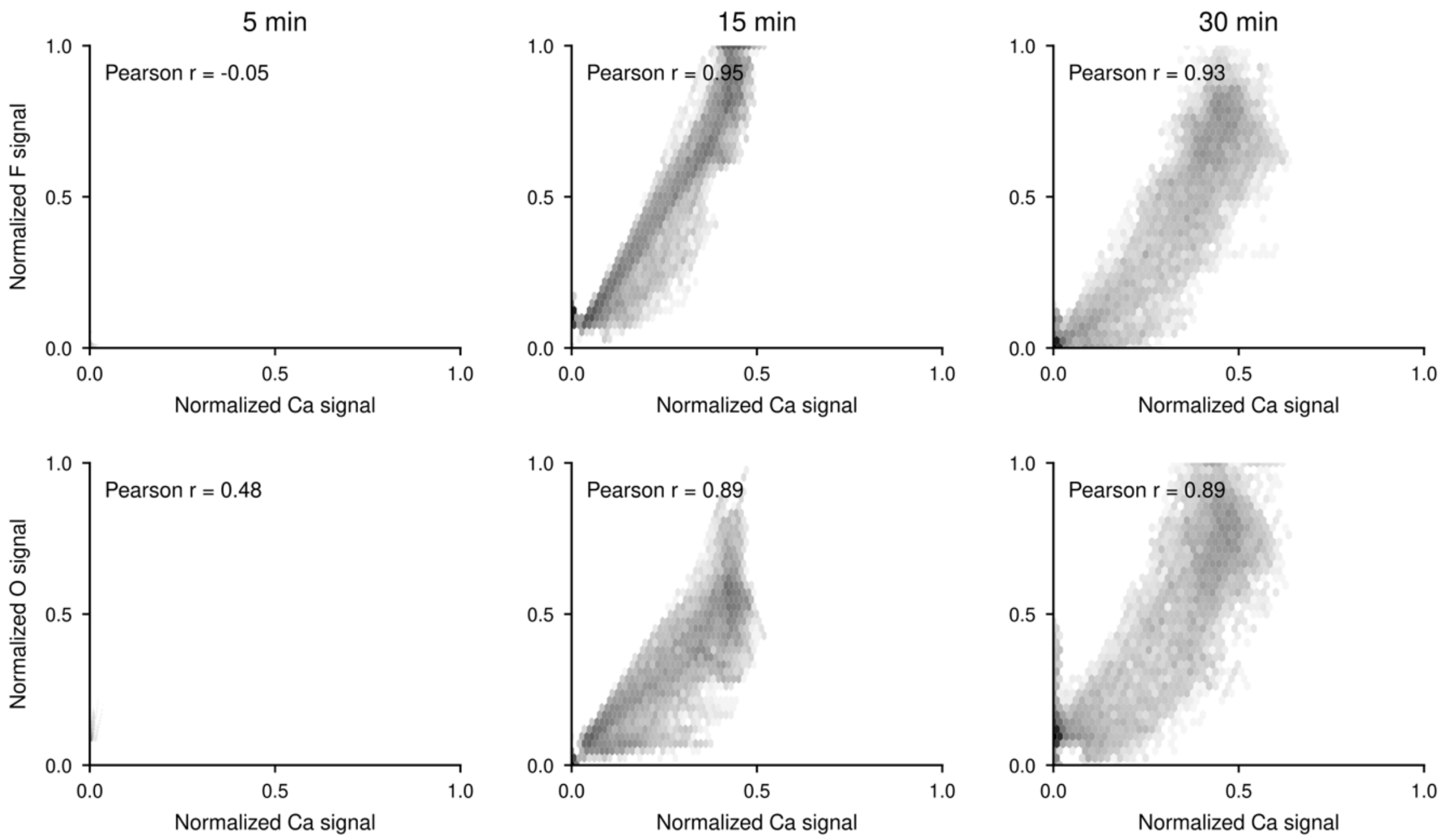

Figure S13. Pixelwise SEM-EDX colocalization analysis of the PTFE + Ca reaction products after 5, 15, and 30 min of milling. Hexagonally binned density plots compare normalized Ca intensity with F (top row) and O (bottom row); darker shading represents a greater number of pixels. Pearson correlation coefficients are shown in each panel. No meaningful Ca-F correlation was resolved at 5 min ($r = -0.05$), whereas strong Ca/F colocalization was observed after 15 and 30 min ($r = 0.95$ and 0.93, respectively). Ca/O colocalization increased from moderate at 5 min ($r = 0.48$) to strong at 15 and 30 min ($r = 0.89$ for both).

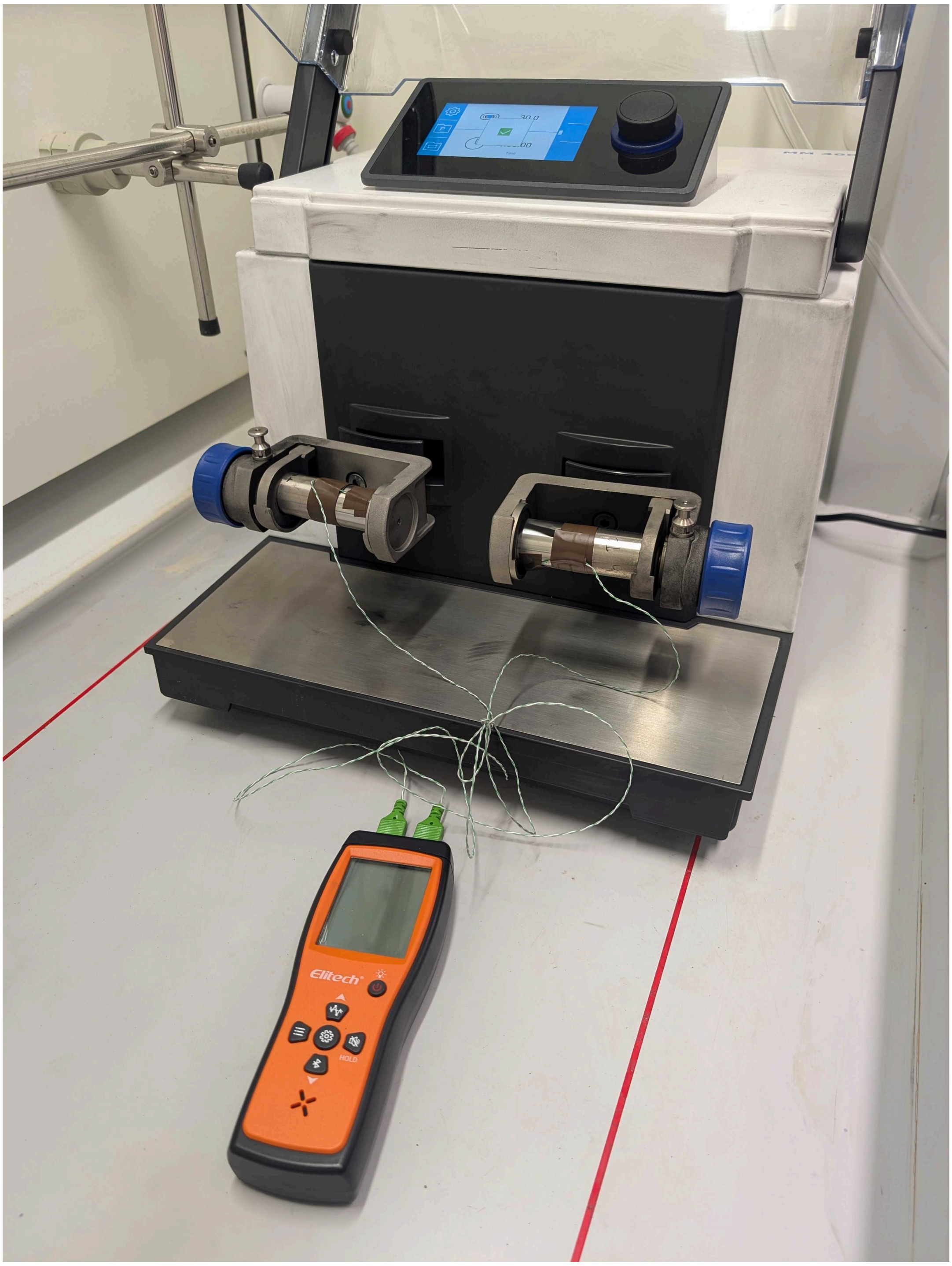

Figure S14. In situ temperature-monitoring setup used in this work. Thermocouples were attached to the milling jars as shown. All reactions were carried out with the safety shield of the mixer mill lowered.

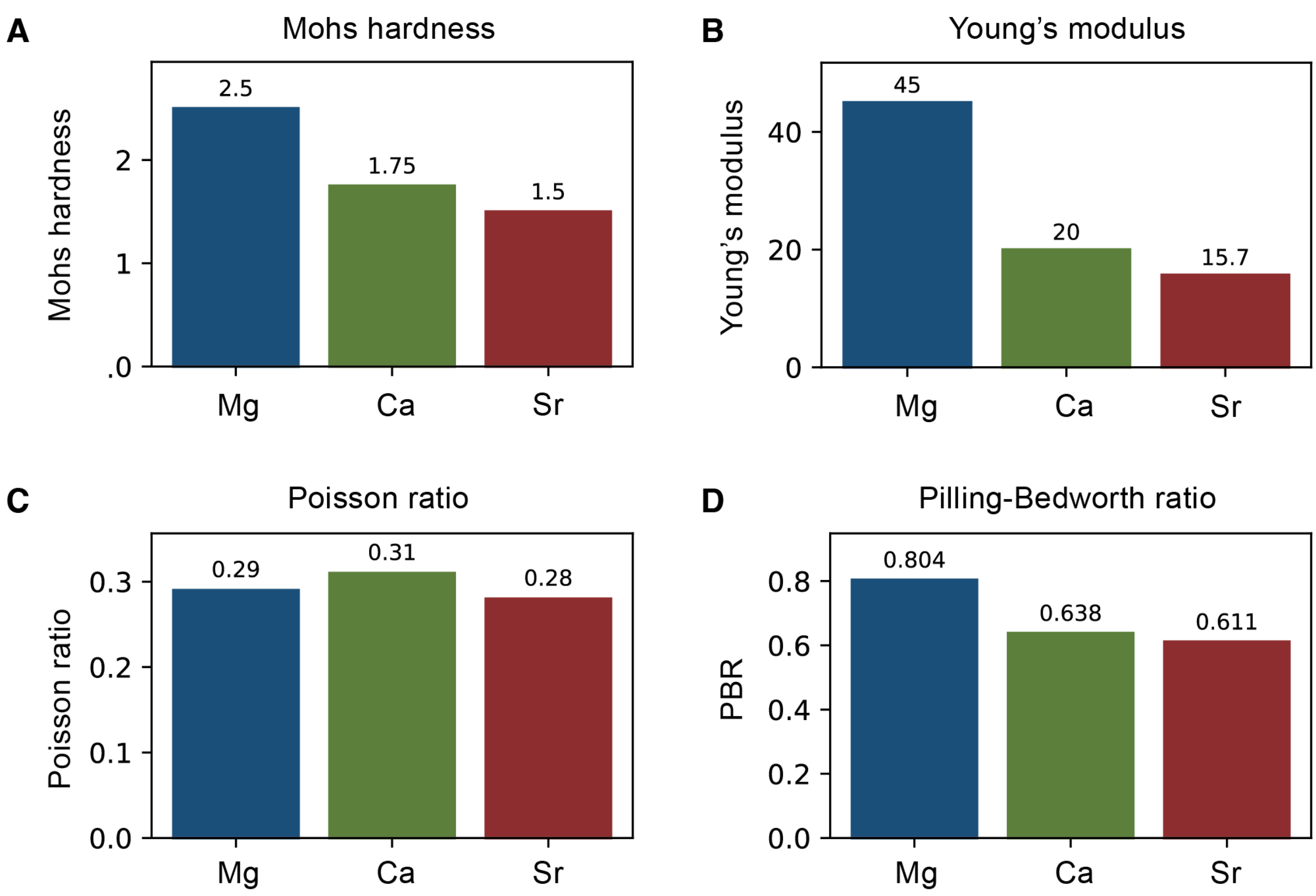

Figure S15. Mechanical properties of the metals. (A) Mohs hardness, (B) Young's modulus (in GPa), (C) Poisson ratio, which describes the transverse deformation of a material in response to axial strain, (D) Pilling-Bedworth ratio, which reflects whether a metal oxide is likely to form a coherent, protective surface layer on the metal surface; for PBR < 1, the oxide layer is too thin to provide protection. Data from references 42 and 43 in the main text.

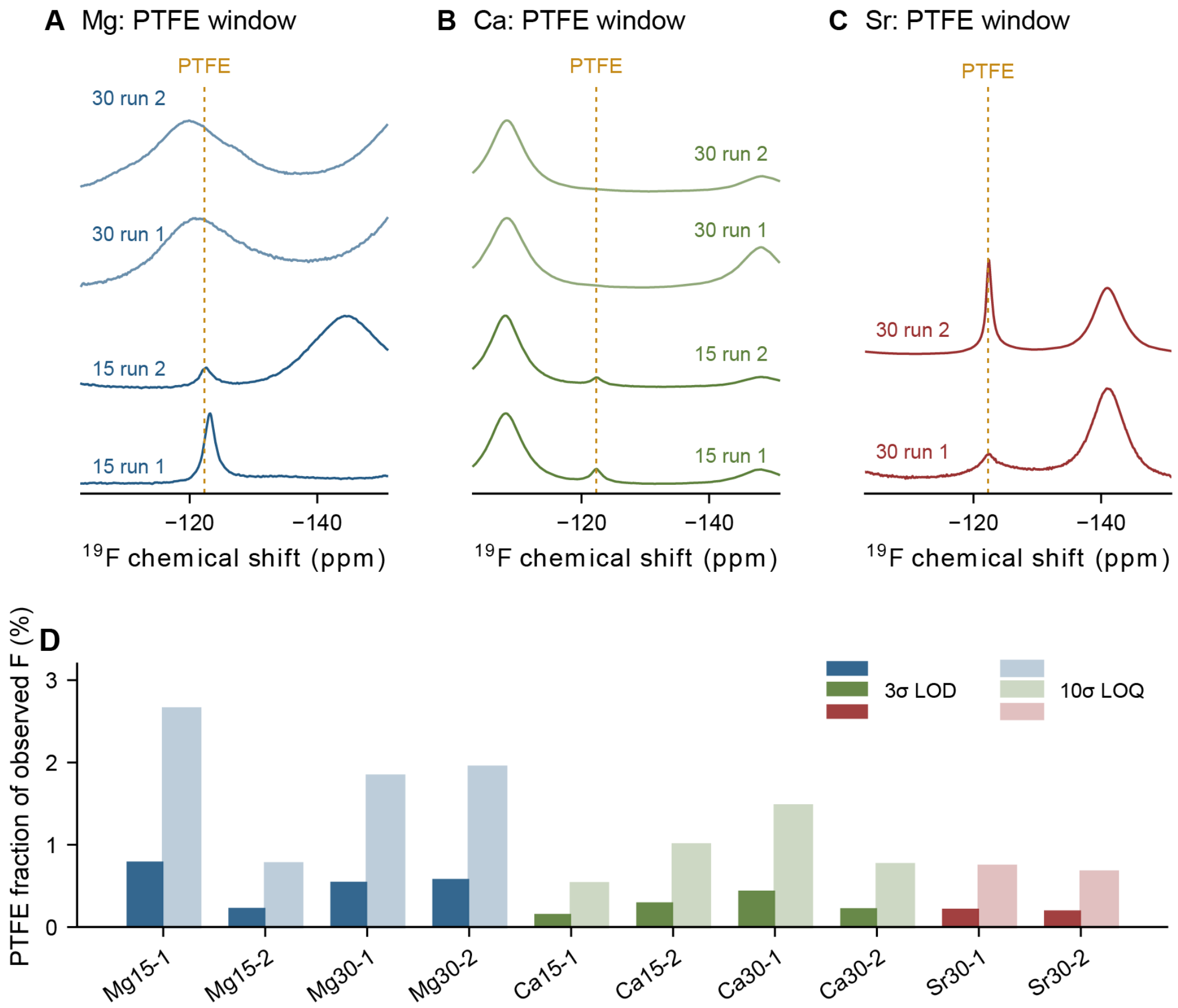

Figure S16. Assessment of the detection limits. PTFE detection windows for the (A) Mg, (B) Ca and (C) Sr reactions, and (D) the 3σ/10σ detection thresholds as explained in Supplementary Note 2. The Mg 30 min non-detections use the conservative 1.0% resolution-limited reporting floor despite a lower statistical LOD.

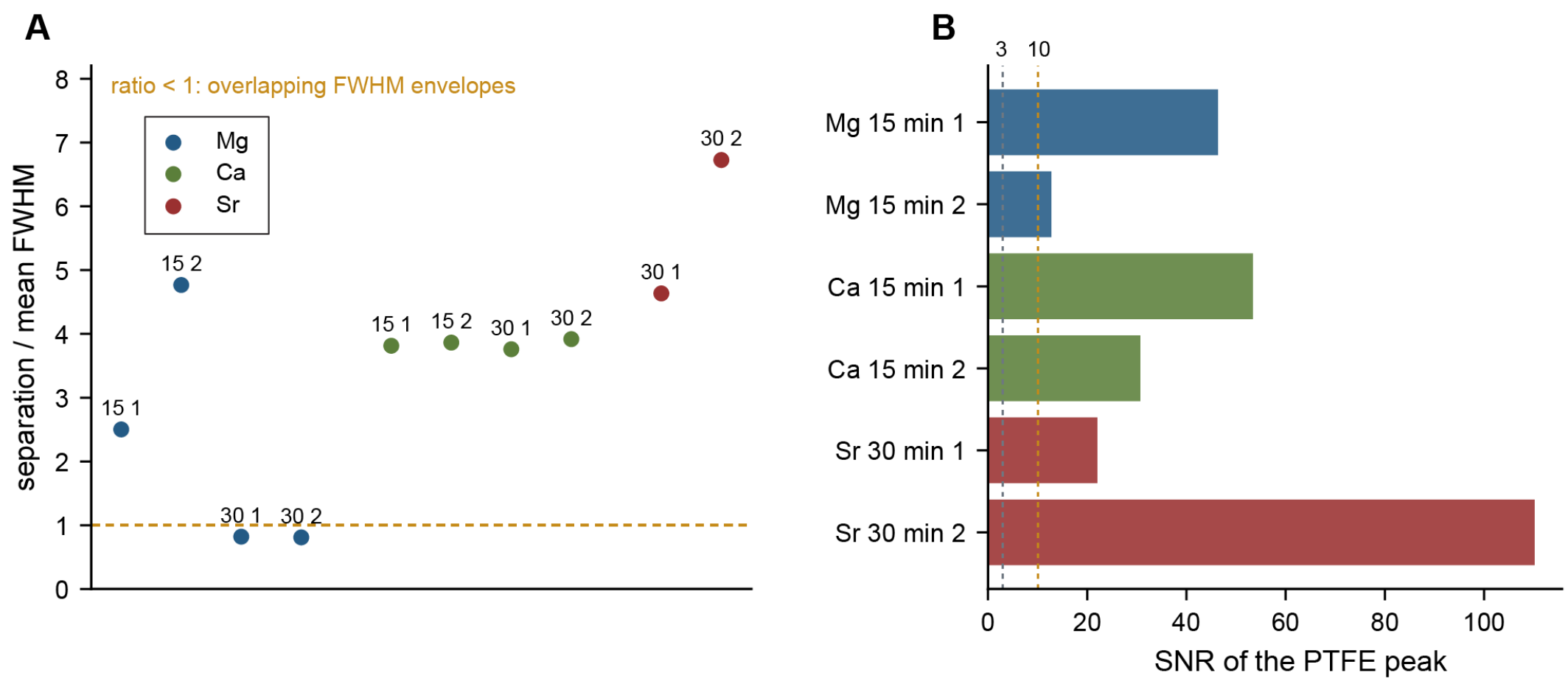

Figure S17. Assessment of resolution and signal-to-noise ratio for quantification of residual PTFE in the ^{19}F MAS NMR spectra. (A) Separation between the expected PTFE isotropic resonance and the nearest metal fluoride resonance or spinning sideband, divided by the mean apparent FWHM of the two features. Values below 1 indicate overlapping signal envelopes. A reference PTFE FWHM of 1.75 ppm was used where no PTFE peak was detected. Labels: first value is the milling time (15 or 30 min), the second value is the run number (1 or 2). (B) Signal-to-noise ratio of the fitted PTFE isotropic peak height (SNR = h_{PTFE}/σ_{noise}) for spectra containing detectable residual PTFE. The robust inter-sideband point-noise estimate (σ_{noise}) is defined in Supplementary Note 2. Dashed lines indicate SNR = 3 and 10.

Table S2. ^{19}F and ^{13}C solid-state NMR acquisition parameters. For stretched T_1 recovery curves, an approximate fitted value is given, indicated with a tilde (~) and the quantitative recycle delay is chosen based on the plateau of the recovery curve, and in a few cases incomplete recovery was corrected according to Supplementary Note 2. For curves that are approximately biexponential, two fitted values are given. n/d - not determined.

Sample	Nucleus	Spin rate (kHz)	Fig.	Pulse sequence	T_1 (s)	Recycle delay (s)	Number of scans	Total time (h)
PTFE	^{19}F	15	2A	echo	~1	1	60	0.02
	^{13}C	15	2B	echo	-	5	160	0.2
PTFE + Mg (30 min, run 1)	^{19}F	15	2A	echo	~3	25	16	0.1
	^{13}C	15	2B	echo	-	5	46904	65
MgF_2	^{19}F	20	2A	echo	0.17	1	16	0.004
PTFE + Ca (30 min, run 2)	^{19}F	15	2A, S1B	echo	~20	100	8	0.2
	^{13}C	15	2B	echo	-	5	30640	43
CaF_2	^{19}F	15	2A	echo	18	90	8	0.2
PTFE + Sr (45 min)	^{19}F	20	2A	echo	~0.4	2	64	0.04
PTFE + Sr (60 min)	^{13}C	10	2B	echo	-	5	12504	17
SrF_2	^{19}F	20	2A	echo	2.3	12	80	0.3
PFOA	^{19}F	15	2A	echo	3.4	5	32	0.04
	^{13}C	15	2B	^{19}F-^{13}C CP	-	2	2064	1.1
PFOA + Ca (30 min, run 2)	^{19}F	15	2A, S1D	echo	0.7; 17	100	16	0.4
	^{13}C	15	2B	echo	-	5	13508	19
PTFE + Mg (30 min, run 2)	^{19}F	15	S1A	echo	0.4; 5	26	44	0.3
PTFE + Mg (15 min, run 2)	^{19}F	20	S1A	echo	~1; 200	1000	32	8.9
PTFE + Mg (15 min, run 1)	^{19}F	12	S1A	echo	~1; 10	100	16	0.4
PTFE + Ca (60 min)	^{19}F	15	S1B	echo	~2	10	3144	8.7
PTFE + Ca (30 min, run 1)	^{19}F	15	S1B	echo	~12	25	16	0.1
PTFE + Ca (15 min, run 2)	^{19}F	15	S1B	echo	8; 101	500	8	1.1
PTFE + Ca (15 min, run 1)	^{19}F	15	S1B	echo	1; 21	100	16	0.4
PTFE + Sr (30 min, run 2)	^{19}F	20	S1C	echo	~2	200	16	0.9
PTFE + Sr (30 min, run 1)	^{19}F	20	S1C	echo	~1	200	256	14.2
PTFE + Ba (30 min)	^{19}F	15	S8B	echo	1.7 (PTFE) 6.1 (BaF_2)	400	8	0.9
PTFE + Ca + heptane (30 min)	^{19}F	15	S9	echo	5.4	25	16	0.11
PFOA + Ca (30 min, run 1)	^{19}F	15	S1D	echo	n/d	100	16	0.4

Table S3. Residual PTFE as a percentage of total relaxation-corrected ^{19}F signal at different milling times. Each run is a separately milled reaction. "<" values are upper bounds, quantified as described in Supplementary Note 2.

	Grinding time (min)	% residual PTFE
PTFE + Mg	0	100
PTFE + Mg, run 1	15	12.5
PTFE + Mg, run 2	15	0.9
PTFE + Mg, run 1	30	<1.0
PTFE + Mg, run 2	30	<1.0
PTFE + Ca	0	100
PTFE + Ca, run 1	15	3.5
PTFE + Ca, run 2	15	4.0
PTFE + Ca, run 1	30	<0.5
PTFE + Ca, run 2	30	<0.3
PTFE + Sr	0	100
PTFE + Sr, run 1	30	3.2
PTFE + Sr, run 2	30	7.4

Table S4. Maximum temperatures and time to peak temperature.

Sample	Maximum T (°C)	Time to peak (s)	Time to peak (min)	Average time to peak (min)
PTFE + Mg, run 1	43.5	656	10.9	13.2
PTFE + Mg, run 2	46.9	926	15.4	
PTFE + Ca, run 1	39.8	366	6.1	6.6
PTFE + Ca, run 2	41.0	421	7.0	
PTFE + Sr, run 1	32.4	235	3.9	4.6
PTFE + Sr, run 2	33.7	320	5.3	

Table S5. Maximum change in temperature (ΔT) and integrated areas under the temperature curves (∫ΔT dt). Baseline temperature is taken as the median of the first 60 seconds of each run.

Sample	Baseline T (°C)	Maximum ΔT (°C)	Average maximum ΔT (°C)	∫ΔT dt (°C·min)	Average ∫ΔT dt (°C·min)
PTFE + Mg, run 1	26.2	17.3	19	188.3	207
PTFE + Mg, run 2	25.9	21.0		225.3	
PTFE + Ca, run 1	23.8	16.0	16	178.4	177
PTFE + Ca, run 2	24.2	16.8		176.5	
PTFE + Sr, run 1	22.6	9.8	10	174.1	175
PTFE + Sr, run 2	22.7	11.0		175.5	
